# Comparison of the pathological response to 2 or 4 cycles of neoadjuvant CAPOX in II/III rectal cancer patients with low/intermediate risks: study protocol for a prospective, non-inferior, randomized control trial (COPEC trial)

**DOI:** 10.1186/s13063-023-07405-x

**Published:** 2023-06-13

**Authors:** Yu Shen, Wanyue Shi, Cui Huang, Xiaoling Gong, Mingtian Wei, Wenjian Meng, Xiangbing Deng, Ziqiang Wang, COPEC groups

**Affiliations:** 1 Colorectal Cancer Center, Department of General Surgery, West China Hospital, Sichuan UniversitySichuan Province, Chengdu, China; 2 Department of Radiology, West China Hospital, Sichuan University, Sichuan Province, Guo Xue Xiang 37#, Chengdu, China

**Keywords:** Rectal cancer, Neoadjuvant chemotherapy, Pathological tumor regression grade, CAPOX

## Abstract

**Background:**

For patients with low- and intermediate-risk stage II/III rectal cancer, current studies have reached a consensus that preoperative radiotherapy may be dispensed with, and neoadjuvant chemotherapy (NCT) alone might achieve an accepted local control. Our previous phase II study has evidenced that the morphological response of NCT could be better judged at a relatively early stage. Low- and intermediate-risk stage II/III rectal cancer patients could achieve a high rate of tumor shrinkage and downgrade after only 4 cycles of NCT and obvious tumor morphological changes could be observed after 2 cycles of NCT. However, there is still a lack of more detailed stratification and evidence for pathological criteria. The aim of the present study (comparison of the pathological response to 2 or 4 cycles of neoadjuvant CAPOX in II/III rectal cancer patients with low/intermediate risks, COPEC trial) is to determine the pathological tumor regression grade (pTRG) rate of 2 or 4 cycles of NCT in low- and intermediate-risk stage II/III rectal cancer and verify the feasibility of early identification of chemotherapy-insensitive population.

**Methods/design:**

This is a multicenter, prospective, non-inferior, randomized controlled trial (RCT) initiated by West China Hospital of Sichuan University and designed to be conducted in fourteen hospitals around China. Eligible patients will be centrally randomized into 2 or 4 cycles of CAPOX in a 1:1 ratio using the central automated randomization system offered by the O-trial online system (https://plus.o-trial.com/) and accept total mesorectal excision after 2 or 4 cycles of CAPOX (oxaliplatin 130 mg/m^2^, once daily on day 1, every 21 days and capecitabine 1000 mg/m^2^, twice daily on days 1 to 14, every 21 days). The primary endpoint is the proportion of patients with pathological no-tumor regression (pTRG 3), which is determined postoperatively by each sub-center and verified by the primary center.

**Discussion:**

COPEC trial is designed to verify that the preoperative CAPOX chemotherapy for low- and intermediate-risk stage II/III rectal cancer could achieve a good response judgment after 2 cycles and obtain the tumor pathological response rate after 2 cycles of CAPOX. We hope the COPEC trial could help in establishing a consensus standard of low- and intermediate-risk rectal cancer and the early identification of stage II/III rectal patients with low- and intermediate-risk who are poorly responding to NCT.

**Trial registration:**

Clinicaltrial.gov NCT04922853. Registered on June 4, 2021.

**Supplementary Information:**

The online version contains supplementary material available at 10.1186/s13063-023-07405-x.

## Background

Neoadjuvant chemoradiotherapy (NCRT) has progressively been accepted as a preoperative treatment for advanced rectal cancer. Many previous large randomized controlled trials (RCTs) [1–4] have shown that NCRT can effectively reduce the local recurrence rate after surgery. NCRT has been recommended as a priority standard treatment for stage II/III rectal cancer by National Comprehensive Cancer Network (NCCN) guidelines [5].

However, despite reducing the local recurrence rate, NCRT has not been proven with improved overall survival and may also bring radio-related adverse effects [4]. Indeed, the introduction of total mesorectal excision (TME) technique, characterized by using sharp dissection along the mesorectal fascia, has reduced the local recurrence rates as low as 5–15% [6, 7]. Local recurrence is no longer the main factor leading to tumor recurrence and death after radical resection. Our previous prospective stratified randomized controlled study [8] divided stage II/III rectal cancer into high-risk and intermediate-low-risk groups according to 5 dimensions (external invasion > 5 mm, lymph node > 8 mm, mesorectal fascia (MRF) ( +), low anterior wall T3, peripheral growth). Both groups were further randomly assigned to the radiotherapy group (receiving short-course radiotherapy with immediate TME surgery) and the surgery group (receiving upfront TME surgery alone). The study showed that the local recurrence rate of rectal cancer in the intermediate-low risk group (whether received radiotherapy or not) was extremely low, with a 3-year cumulative local recurrence rate of 3% [8], indicating that at least part of patients with intermediate-low risk was less likely to benefit from radiotherapy, which was also similar to other studies [9–15]. At the same time, radiotherapy did not bring a significant 5-year survival benefit in either group.

Although current studies suggest that patients with low- and intermediate-risk stage II/III rectal cancer could consider dispensing with preoperative radiotherapy, and NCT alone might achieve an accepted local control, there is still a lack of more detailed stratification and evidence for pathological criteria. For this reason, we have conducted a phase II study that included low- and intermediate-risk stage II/III rectal cancer for NCT alone and found that the proportion of patients with tumor shrinkage and downgrade after only 4 cycles of chemotherapy for low- and intermediate-risk stage II/III rectal cancer was 78.7%, with the pCR rate of 21.3% [16]. We also found that the 4 cycles of CAPOX were effective, and obvious tumor morphological changes were observed after 2 cycles (duration 6 weeks), with a predicted AUC value of 0.862 (0.751, 0.973). These findings suggested that the morphological response of NCT could be better judged at a relatively early stage. Therefore, we tended to hold the view that a considerable number of patients would be able to achieve tumor regression, and the pathological effect would reach tumor regression grade (TRG) 2 or better results after two cycles of NCT.

Currently, there is still no reliable preoperative method of assessing tumor response after NCT [17, 18]. Based on our previous phase II study, to verify that the CAPOX chemotherapy for low- and intermediate-risk stage II/III rectal cancer could achieve a good response judgment after 2 cycles, and obtain the tumor pathological response rate in the early 2 cycles, we intend to conduct a prospective, non-inferior, randomized, controlled study (COPEC trial) to determine the pathological tumor regression grade (pTRG) rate of 2 or 4 cycles of NCT in low- and intermediate-risk stage II/III rectal cancer and explicit the feasibility of early identification of chemotherapy-insensitive population.

## Methods/design

### Objective

The primary objective of this study is to prospectively evaluate that the pathological response rate after 2 cycles of CAPOX is not inferior to that of 4 cycles in patients with low-risk and intermediate-risk stage II/III rectal cancer, which may provide evidence for the feasibility of early identification of chemotherapy-insensitive population.

The secondary objectives are as follows: (1) to explore the difference in clinical remission rate between 2 and 4 cycles of CAPOX in patients with low-risk and intermediate-risk stage II/III rectal cancer; (2) to explore the effectiveness/ineffectiveness of NCT at early cycles (after 2 cycles of CAPOX) in predicting the long-term prognosis (3 years overall survival (OS)/disease-free survival (DFS)); (3) to confirm the perioperative safety of NCT alone; (4) to construct a model using 2-cycle imaging indicator to predict a 4-cycle pathological remission; and (5) to explore the feasibility of artificial intelligence (AI)-assisted imaging indicators to predict pTRG after NCT.

### Patients and study design

This is a multicenter, prospective, non-inferior, randomized controlled study initiated by West China Hospital of Sichuan University and designed to be conducted in fourteen hospitals around China (Additional file 1).

**Fig. 1:**
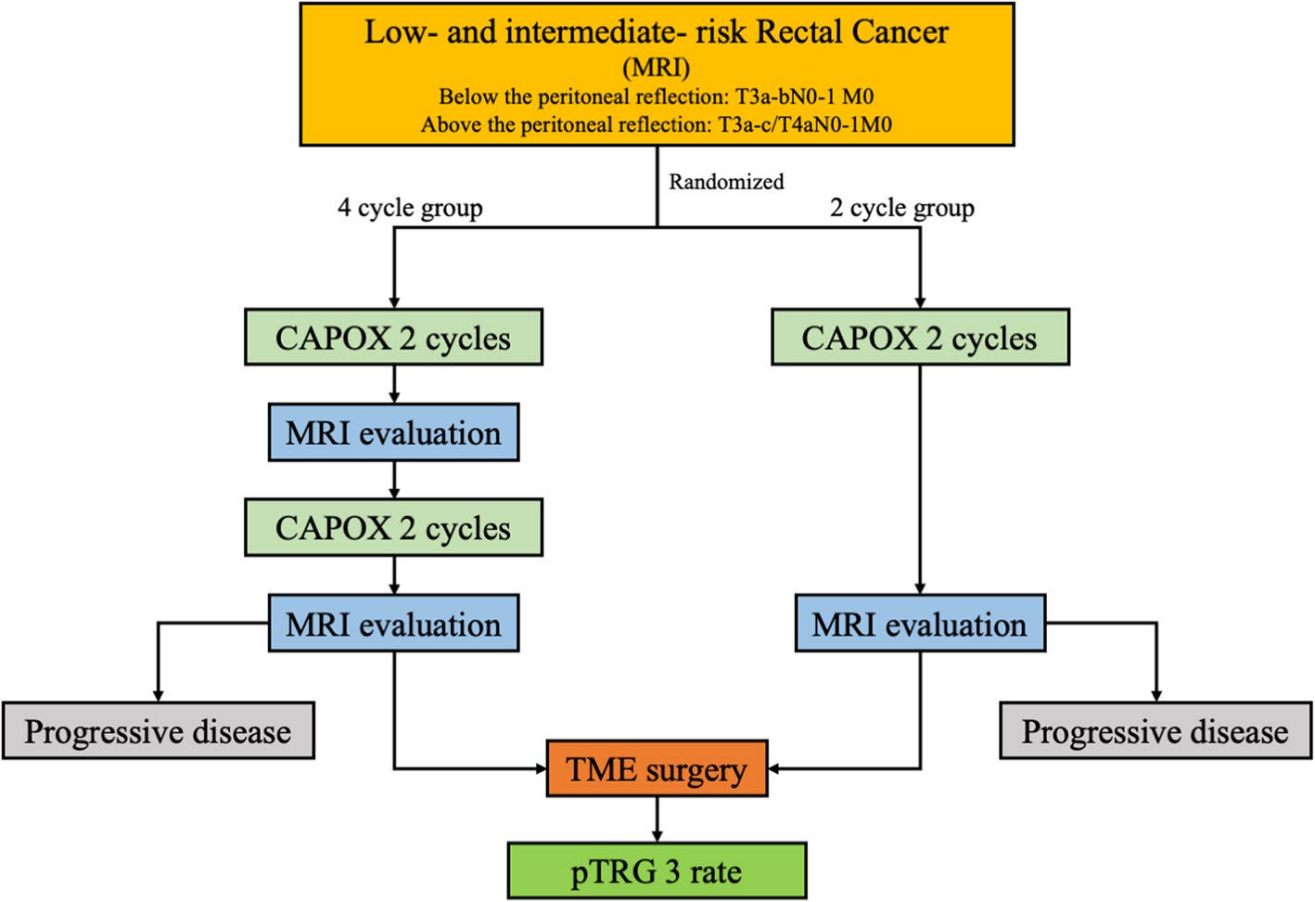
Flow diagram

Patients with low-risk and intermediate-risk stage II/III rectal cancer are designed to be included in the study. The flow diagram has been shown in Fig. 1. In brief, patients diagnosed with rectal cancer will be evaluated by enhanced abdominal CT, pelvic MRI, and transrectal ultrasonography (TRUS) and staged according to the European Society for Medical Oncology (ESMO) guidelines [19]. Eligible patients will be randomized to accept 2 or 4 cycles of pre-preoperative CAPOX. Re-evaluation will be performed after 2 cycles of CAPOX (2 and 4 cycles group) and 4 cycles of CAPOX (4 cycles group). MrTRG will be recorded and verified by pTRG after TME surgery. Detailed criteria for mrTRG and pTRG have been listed in Additional files 2 and 3, respectively.

Low- and intermediate-risk stage II/III rectal cancer was defined as (1) middle and low rectal cancer (below the peritoneal reflection): T3a-bN0-1M0, EMVI ( ±), MRF (-) (≥ 2 mm) and middle and high rectal cancer (above the peritoneal reflection): T3a-c/T4aN0-1M0, EMVI ( ±), MRF (-) (≥ 2 mm); (2) mesorectal lymph node with short diameter ≥ 8 mm or highly suspected metastasis not more than 3; and (3) the short diameter of the lateral lymph node ≤ 7 mm. Inclusion, exclusion, and withdrawal criteria have been detailed in Table 1. The screening process is set to be performed by experienced surgeons at each center. Enrolled patients are allowed to quit at any time without any reason or responsibility.

**Table 1:** Details of the inclusion, exclusion, and withdrawal criteria

Inclusion criteria	
1)	Patients aged between 18 and 75 of either sex
2)	Patients with low- and intermediate-risk stage II/III rectal cancer evaluated by MRI and TRUS: middle and low rectal cancer (below the peritoneal reflex line): T3a-bN0-1M0, EMVI ( ±), MRF (-) (≥ 2 mm) and middle and high rectal cancer (above the peritoneal turning line): T3a-c/T4aN0-1M0, EMVI ( ±), MRF (-) (≥ 2 mm) Mesangial lymph node with short diameter ≥ 8 mm or highly suspected metastasis no more than 3 The short diameter of the lateral lymph node ≤ 7 mm Patients with ultra-low rectal cancer who match the above criteria and can achieve negative circumferential resection margin under extralevator abdominoperineal excision (ELAPE) surgery will be included in the group
3)	In colonoscopy or anal examination, the lower boundary of the lesion is ≤ 12 cm from the anal verge
4)	Patients with no distant metastasis (including suspected lung nodule metastasis) confirmed by chest and abdomen CT examination; no extra-regional lymph node metastasis (≥ 10 mm)
5)	Pathologically diagnosed rectal adenocarcinoma
6)	Eastern Cooperative Oncology Group (ECOG) score: 0–1
7)	Patients with primary rectal cancer who have not received surgery (except palliative ostomy), radiotherapy, systemic chemotherapy, or other anti-tumor treatments before enrollment
8)	The main organ function is normal and meets the following standards: (1) blood routine criteria: HB ≥ 9 g/dL, WBC ≥ 3.5/4.0 × 10^9^/L, neutrophil ≥ 1.5 × 10^9^/L, PLT ≥ 100 × 10^9^/L; (2) blood biochemical criteria: Crea and BIL ≤ 1.0 times the upper limit of normal value (ULN), ALT and AST ≤ 2.5 times the upper limit of normal value (ULN), alkaline phosphatase (ALP) ≤ 2.5 × UNL, total bilirubin (Tbil) ≤ 1.5 × UNL
9)	No history of 5-Fu and platinum drug allergy
10)	Females of childbearing age must undergo a pregnancy test (serum or urine) 7 days before enrollment with a negative result and are willing to use appropriate contraceptive methods during the trial and 8 weeks after the last administration
11)	Patients voluntarily join the study and sign informed consent forms with good compliance and follow-up
Exclusion criteria joined	
1)	Patients considering lynch syndrome
2)	Patients who do not consider metastasis in the initial diagnosis but proved to be distant metastases during the treatment
3)	Previously or concurrently suffering from other malignant tumors (including concurrent colon cancer), except for cured skin basal cell carcinoma and cervical carcinoma in situ
4)	Pregnant or nursing women
5)	Lateral lymph nodes ≥ 7 mm
6)	Patients with severe cardiovascular disease and diabetes difficult to control
7)	Patients with mental disorders
8)	Patients with severe infection
9)	Patients undergoing thrombolysis/anticoagulation therapy with bleeding diathesis or coagulation dysfunction, or suffering aneurysm, stroke, transient ischemic attack in the past year
10)	Patients with a history of kidney disease, urinary protein, or clinically abnormal renal function
11)	Patients with a history of gastrointestinal fistula, perforation, bleeding, or severe ulcer
12)	Patients with severe gastrointestinal diseases that affect the absorption of oral chemotherapy drugs
13)	Patients participating in another clinical trial within 4 weeks before treatment
14)	Patients pathologically diagnosed with mucinous component or the signet-ring cell carcinoma
Withdrawal criteria	
1)	Patients refusing further treatment
2)	Patients having severe adverse reactions to chemotherapy and being unable to complete 2 cycles of chemotherapy

### Randomization and intervention

Each sub-center will be responsible for potential enrollment and consent-taking. Central randomization will be performed as soon as the enrollment is determined using the central automated randomization system offered by the O-trial online system (https://plus.o-trial.com/). Sub-center will upload the information of eligible patients on the O-trial system, which will sequentially number and randomize the uploaded patients into 2 or 4 cycles of CAPOX in a 1:1 ratio automatically. No stratification is included in the current study.

Patents in both groups will undergo TME surgery after 2 or 4 of CAPOX. Each cycle of CAPOX includes oxaliplatin 130 mg/m^2^, once daily on day 1, every 21 days and capecitabine 1000 mg/m^2^, twice daily on days 1 to 14, every 21 days. TME surgery was performed by experienced colorectal surgeons 2–3 weeks after the last cycle of preoperative CAPOX.

Postoperative adjuvant treatment will be performed according to the ESMO and Chinese guidelines for colorectal cancer [19, 20]. Based on the pathological stage, patients with a positive CRM or N2 stage would be recommended to undergo postoperative radiotherapy. Adjuvant chemotherapy was recommended for N + patients. The long-term survival of the patients will be followed up.

### Data collection

Clinical, imaging, and follow-up data will be collected prospectively in the case report forms (Additional file 4) and reserved in the O-trial online database (https://plus.o-trial.com/), which is daily maintained by WY, Shi and C, Huang. The O-trial online database is a secure, password-protected database and can be only accessible to authorized project staff.

Details of the evaluation items and time points have been shown in Table 2. During CAPOX, blood routine and biochemistry will be examined weekly to detect potential hematopoietic inhibition or liver or kidney function impairment. Tumor markers, at least including CEA and CA19-9, will be recorded every cycle. Imagological examination, including chest CT, abdominal enhanced CT, rectal MRI, and TRUS will be re-evaluated every 2 cycles.

**Table 2:** Details of the evaluation items and time points

	Screening	After 2 cycles of CAPOX (4 cycles)	Preoperative evaluation	Intraoperative evaluation	Postoperative evaluation	Follow-up visit
Colonoscopy	×					
Chest CT	×	×	×			×
Abdominopelvic enhanced CT	×	×	×			×
Pelvic MRI	×	×	×			
TRUS	×	×	×			
Blood routine	×	×	×			×
Blood biochemical indicators	×	×	×			×
Tumor markers^a^	×	×	×			×
Intraoperative complications				×		
Blood loss				×		
Operation time				×		
Surgical approach				×		
Perioperative complications					×	
Patient recovery					×	
Hospitalization time					×	
pTRG					×	

*TRUS* Transrectal ultrasonography, *pTRG* Pathological tumor regression grade

^a^At least includes CEA and CA19-9

Surgical information including tumor location and involvement, intraoperative stages, lymph node metastasis, operation times, intraoperative time, and complications will be recorded in detail in the surgical records. Perioperative complications are stratified by the Clavien-Dindo system. Resected rectal specimens will be fixed on a foam plate and sent to pathologists to determine the circumferential resection margin (CRM) and pTRG).

Follow-up is set throughout the whole trial (Additional file 5). In the preoperative CAPOX process, included patients will be asked to finish a series of questionnaires about quality of life (QOL) and tumor or NCT-related symptoms each cycle. Postoperative follow-up is set at 3 months after surgery, every 6 months in the first 5 years, and every year in subsequent years. QOL, blood tests, and CT imaging are also included in each follow-up. Adverse effects of postoperative adjuvant chemotherapy will also be recorded.

QOL is measured based on EORTC QLQ-C30 [21, 22], the EORTC QLQ-CR29 questionnaire [23], the International Prostatic Symptom Score (I-PSS) [24], and the sexual function evaluation questionnaire, which will be gathered by physician visits, mail, or WeChat and recorded in the online system for further analysis. Radiological examinations will be individually assessed by two radiologists blinded to the groups. Tumor imaging data, such as tumor long diameter, tumor volume, ADC value, and DWI value, will be collected and mrTRG will be determined accordingly. When a discrepancy arose, the imaging scans will be reviewed by two experienced radiologists in West China Hospital to reach a consensus. Based on MRI imaging, tumor response to NCT is classified into grades 1–5 (complete, good, moderate, slight and no response), and mrTRG4 and mrTRG5 represent a poor response to NCT. Criteria of the mrTRG have been listed in Additional file 2. Pathology examinations are performed by each sub-center and verified by the primary center to determine the pTRG (0–3). A pTRG3 represents poor tumor response to NCT. Specific standards of pTRG have been shown in Additional file 3.

### Endpoints

The primary endpoint is the proportion of patients with pTRG3, which is determined postoperatively by each sub-center and verified by the primary center.

The secondary endpoints include (1) the proportion of patients with TRG on MRI (mrTRG) (1–5) after 2 cycles/4 cycles of CAPOX; (2) the proportion of patients with pTRG (0–2); (3) survival prognosis of patients with different pTRG, containing disease-free survival (DFS), local recurrence rate (LR), distant recurrence rate (DR), and overall survival (OS); (4) 3-year DFS and OS of patients with different pTRG; (5) the predicted imaging indicators of pTRG; (6) the accuracy of pTRG judged by imaging; (7) the adverse effects of chemotherapy; (8) the incidence of perioperative complications; and (9) the QOL of patients during chemotherapy and after surgery.

### Sample size calculation

According to phase II trial [16], the proportion of patients who had poor pathological remission (pTRG 3) after 4 cycles of CAPOX was 27.9%. The proportion of pTRG3 after 2 cycles of CAPOX is expected to increase by no more than 10%. The non-inferiority test is used with *α* = 0.05, (1-β) = 0.8, and the non-inferiority margin (hazard ratio) value δ is set as 0.05. Pearson chi-square test is used to calculate the sample size required for the experiment, and 249 samples are required for each group calculated by a normal approximation algorithm [25]. Ten percent of patients are estimated to withdraw from the group or lost to follow-up; 277 patients are needed in each group. The planned enrollment time is 2.5 years, and the total number of patients is about 554 cases. Meanwhile, it is assumed that the dropout rate of patients in the 4 cycles of the CAPOX group is about 6% due to intolerance of chemotherapy. Therefore, it is estimated that 294 patients would be included in the 2 cycles of the CAPOX group and 260 patients in the 4 cycles of the CAPOX group.

### Data-analysis

The trial statistician, who will be blinded to randomization, will carry out the data analysis process. Before analysis, they will assess the integrity of collected data, and repeated or missing data will be described accordingly. Demographics and repeatedly measured clinical characteristics of participants will be summarized as the baseline.

SPSS for Windows, version 25 (IBM Corp, Armonk, NY), and GraphPad Prism for Windows, version 8.0.0 (GraphPad Software, San Diego, California), are used for data analysis. Continuous data are shown as the mean and standard deviation. Categorical variables are represented as percentages. Continuous variables are compared using the Mann–Whitney *U* test. Categorical variables are compared between groups using Fisher’s exact test or the chi-square test. Fisher’s exact test or the chi-square test is used to compare the proportions of patients with pTRG3, the proportion of patients with TRG on MRI (mrTRG) (1–5) after 2 cycles/4 cycles of CAPOX, and the proportion of patients with pTRG (0–2). Survival of patients (OS, DFS) is compared using Kaplan–Meier analysis. Statistical significance is defined as a *p*-value less than 0.05. Especially, in the 4 cycles of the CAPOX group, patients who receive less than 3 cycles of chemotherapy due to adverse effects or drop-out will be analyzed as the 2 cycles of the CAPOX group; others will be analyzed in the 4 cycles of the CAPOX group analysis.

### Ethics and safety

The trial has been approved by the Ethics Committee of the West China Hospital, Sichuan University (2021 No. 376), and registered on ClinicalTrials.gov (registration number NCT04922853). Each participant has signed an informed consent form (ICF, Additional file 6) before enrollment. We will request consent for the review of participants’ medical records and for the collection of blood samples to detect potential hematopoietic inhibition or impairment of liver or kidney function, and tumor markers, at least including CEA and CA19-9, which will be recorded every cycle. Resected rectal specimens will also be stored for pathological review. Participants have the right to quit the study at any time without any reason.

### Monitoring and reporting

Information of the coordinating center has been listed in Additional file 1. Each sub-center will be responsible for identifying potential patients and taking their consent independently, while center randomization will be made subsequently after inclusion by West China Hospital of Sichuan University. Also, each sub-center is responsible for monitoring the safety of their included patients, and the West China Hospital of Sichuan University is responsible for the overall COPEC trial. The trial steering committee (TSC) is composed of professional investigators of each sub-center and is responsible for supervising the trial. The regular meeting of TSC is set every 6 months to oversee conduct and progress.

Patients are covered by health insurance in case the adverse effects from NCT occur and will be withdrawn from the trial if they are evidenced sufferings risks beyond expected based on medical reasons. Detected serious adverse events will be submitted to the related medical ethical committee for further evaluation within 1 month after the occurrence. All adverse events will be updated on ClinicalTrials.gov. Investigators can examine the detail of the adverse events online.

Adverse events mainly include tumor progression and serious chemotherapy adverse effects during CAPOX. Tumor progression is defined as a tumor longitudinal size increase greater than 20%. Patients will be advised to withdraw from the trial if the tumor is determined a progressive disease (PD). The chemotherapy-related adverse effect is graded according to CTCAE 5.0, and adverse effects graded 3–4 are defined as serious adverse effects. The occurrence and recording of adverse reactions are responsible for the sub-center and follow the principles below:

1. If a 1/2° adverse reaction occurs, no special treatment is required.
2. Capecitabine/oxaliplatin should be discontinued and corresponding symptomatic treatment should be given when 4° leukopenia, 3° adverse reactions of the digestive tract, 2° anemia and thrombocytopenia, and 2° liver and kidney function impairment occur. If the adverse reactions decreased to 0 ~ 1° within 5 days after treatment, the original dose of capecitabine/oxaliplatin can be restored. Otherwise, capecitabine/oxaliplatin should be reduced to 75%. Capecitabine/oxaliplatin treatment should be discontinued if these adverse reactions persist for more than 3 days after symptomatic treatment and dosage reduction or other adverse reactions above 2° occur.
3. When other 3° adverse reactions occur, the treatment principle is the same as the corresponding 2° adverse reactions.
4. Chemotherapy should be stopped in case of any 4° adverse reactions other than leukopenia occurring.

### Patient and public involvement

Patients or the public are excluded from the design of the trial and the selection of endpoint parameters since the determination of tumor stage and decision of whether NCT should be implemented is a crucial step that needs professional backgrounds. Patients are excluded from the recruitment and conduct of the trial either. The schedule of the current study has been listed in Additional file 5. The results of the present study will be published via medical journal(s). Participants of the study will be informed of the outcomes by local monitors via phone or letter.

### Protocol amendments

Substantive amendments to the current version of the protocol will be detailed and submitted to the institutional review board committee at the West China Hospital, Sichuan University, for approval. We will also update the amendments on ClinicalTrials.gov.

## Discussion

NCRT has been used widely to improve both local control and outcomes in patients with rectal cancer, but it also brought side effects related to the therapy.

Given our previous phase II clinical study found that NCT alone achieved good tumor response rates in patients with low- and intermediate-risk stage II/III rectal cancer, and predicting tumor response to NCT was feasible at an early treatment phase, we conducted this prospective, non-inferior, randomized, controlled study (COPEC trial) to verify the tumor pathological response rate in patients with 2 cycles of CAPOX was not inferior to that of 4 cycles and the determination of chemotherapy-insensitive population at early cycles is feasible.

For rectal cancer (RC), stratified treatment has been increasingly recommended and included in ESMO guidelines [19]. However, the criteria of stratification still need to be further explored, and the curative effect of each stratification has still not been determined. For high-risk rectal cancer, NCRT has been proven with reduced local recurrence rate, although improved overall survival has not been proven. For intermediate-low-risk rectal cancers, our previous study has found that patients diagnosed with stage II/III rectal cancers without high-risk factors were less likely to benefit from radiotherapy. At the same time, radiotherapy did not bring significant 5-year survival benefits. Therefore, a consensus has been reached that patients with low- and intermediate-risk stage II/III rectal cancer could consider dispensing with preoperative radiotherapy, and chemotherapy alone might achieve an accepted local control.

For distant control of tumor cells, FOWARC, GRECCAR 4, and other trials [26–29] explored whether radiotherapy-free neoadjuvant chemotherapy alone could improve patients prognosis and have observed the pathologic complete response (pCR) ranging from 7 to 12.2%. These studies included patients with stage II/III rectal cancer in different strata, without specific stratification for risk factors. In the exploration of combined targeted therapy, studies showed that in patients with advanced stages who have risk factors such as ≥ T3c, N2, MRF ( +), etc., even if targeted drugs were used in combination, the pCR rate was still lower by 13% [30] and 4% [31]. However, if only patients with low- and intermediate-risk factors were included in targeted therapy, the pCR rate could reach 25% [32] and 20% [33]. A study of the National Cancer Center of Japan found that the local recurrence rate of high-risk rectal cancer patients with NCT alone was as high as 19.6%, and the local recurrence rate of those with poor response to chemotherapy was as high as 34% with a low survival rate [34]. The PROSPECT study included relatively low-risk (non-T4, N2) stage II/III rectal cancer patients [34] and obtained a pCR rate of 25%, despite some high-risk T3c-d in T3 population [35] being included. Therefore, current studies suggest that preoperative NCT alone may benefit patients with low- and intermediate-risk stage II/III rectal cancer, but there is still a lack of more detailed stratification and evidence for pathological criteria. For this reason, we have conducted a phase II study which included low- and intermediate-risk stage II/III rectal cancer for NCT alone and found that the proportion of patients with tumor shrinkage and downgrade after only 4 cycles of NCT for low- and intermediate-risk stage II/III rectal cancer was 78.7%, with the pCR rate of 21.3% [16].

At present, there are retrospective and small prospective studies [27, 28, 32, 34, 36] that have shown that chemotherapy alone could still achieve an excellent prognosis for low- and intermediate-risk stage II/III rectal cancer, and there are also large RCTs that are validating whether neoadjuvant chemotherapy alone non-inferior to neoadjuvant chemoradiotherapy for low-risk stage II/III rectal cancer [37]. Although the results have not yet been announced, it is speculated from the existing evidence that NCT alone can achieve relatively good results for patients with low- and intermediate-risk. However, the evaluation time of chemotherapy effects in previous studies was 12 weeks after chemotherapy (6 cycles of FOLFOX or 4 cycles of CAPOX), which was relatively long. Patients who are not sensitive to chemotherapy may need to convert to surgery, radiotherapy, and other intensive treatments in advance, and the relatively long-term chemotherapy may lead to delay of patients’ opportunity to receive necessary treatment, causing a series of oncology consequences and acquiring unnecessary adverse effects of chemotherapy.

The power of the COPEC trial lies in its 2 verse 4 cycles’ design to acquire early pathological outcomes after NCT. Based on our previous phase II study, we hypothesize COPEC trial could obtain a good pathological response judgment in patients with low- and intermediate-risk stage II/III rectal cancer after 2 cycles of preoperative CAPOX chemotherapy. The major challenge of the COPEC trial is the difficulty in patient enrollment, which is mainly due to the worry that 2-cycle CAPOX maybe not be powerful enough in dealing with local advanced rectal cancer. However, we have proved in the previous phase II study that obvious tumor morphological changes were observed after 2 cycles of preoperative CAPOX chemotherapy and those who responded poorly after 2 cycles of NCT would not benefit from 2 more cycles. So perhaps, in the future, the follow-up standard treatment plan for patients with low- and intermediate-risk rectal cancer may become NCT alone without radiotherapy combined with early assessment of tumor response and screening out those with poor response to NCT for further advanced treatment. We hope the COPEC trial could help in establishing a consensus standard of low- and intermediate-risk rectal cancer and the early identification of stage II/III rectal patients with low- and intermediate-risk who are poorly responding to NCT.

## Trial status

This paper reflects protocol version 1.2. dated 6 December 2021. The trial was first registered on ClinicalTrials.gov on 4 June 2021, with the last update posted on 16 March 2022 (NCT04922853). Participant recruitment began on 6 June 2021 and is expected to be completed in December 2023. Raw data are expected to be available for publication after the study via an open-access database.

## Supplementary Information

**Additional file 1. **List of coordinating center.

**Additional file 2: Supplementary Table 1.** Criteria of the magnetic resonance tumor regression grade (mrTRG).

**Additional file 3: Supplementary Table 2.** Criteria of the pathological tumor regression grade (pTRG).

**Additional file 4. **Case Report Form (CRF).

**Additional file 5. **Schedule Of Enrolment, Interventions, And Assessments.

**Additional file 6. **Informed Consent.

## Data Availability

The study co-primary investigators (Ziqiang Wang and Wenjian Meng) will provide the full study protocol, study data, and statistical code upon reasonable request and approval of an appropriate data-sharing agreement.

## Abbreviations

RC: Rectal cancer
RCTs: Randomized controlled trials
NCRT: Neoadjuvant chemoradiotherapy
NCT: Neoadjuvant chemotherapy
TME: Total mesorectal excision
AI: Artificial intelligence
NCCN: National Comprehensive Cancer Network
ESMO: European Society for Medical Oncology
MRF: Mesorectal fascia
pCR: Pathologic complete response
MRI: Magnetic resonance imaging
CT: Computed tomography
TRUS: Transrectal ultrasonography
CEA: Carcinoembryonic antigen
CA19-9: Carbohydrate antigen 19–9
TRG: Tumor regression grade
mrTRG: MRI tumor regression grade
pTRG: Pathological tumor regression grade
DWI: Diffusion-weighted imaging
ADC: Apparent diffusion coefficient
DFS: Disease-free survival
OS: Overall survival
MRD: Molecular residual disease
Life quality (EORTC-QLQ-C30): This sub-questionnaire contains the 2 items of the global quality of life dimension of the EORTC-QLQ-C30 questionnaire
Life quality (EORTC-QLQ-CR29): This questionnaire is developed to assess the quality of life in colorectal patients
I-PSS: International Prostatic Symptom Score
QOL: Quality of life
CTCAE: Common Terminology Criteria for Adverse Events
CRM: Circumferential resection margin
LR: Local recurrence rate
PD: Progressive disease
DR: Distant recurrence rate
ICF: Informed consent form
