# Supplementary material for: Comparison of the pathological response to 2 or 4 cycles of neoadjuvant CAPOX in II/III rectal cancer patients with low/intermediate risks: study protocol for a prospective, non-inferior, randomized control trial (COPEC trial)
Source: Trials. 2023 Jun 13;24:397. doi: 10.1186/s13063-023-07405-x (PMC10262432; doi:10.1186/s13063-023-07405-x)
Supplement: Supplementary file 1 — Additional file 1. List of coordinating center. [file 13063_2023_7405_MOESM1_ESM.docx]

List of the coordination center

1. West China Hospical, Sichuan University

2. Peking Union Medical College Hospital

3. Beijing Cancer Hospital

4. Sun Yat-sen University Cancer center

5. The second Affiliated Hospital of Zhejiang University School of Medicine

6.Yunnan Cancer Hospital

7. The First Affiliated Hospital of Zhengzhou University

8. The Affiliated Hospital of Guizhou Medical University

9. People’s Hospital of Sichuan Province

10. The Third People’s Hospital of Chengdu

11. The Fifth People’s Hospital of Chengdu

12. Nanchong Center Hospital

13. Dazhou Center Hospital

14. People’s Hospital of Leshan
