# Supplementary material for: Comparison of the pathological response to 2 or 4 cycles of neoadjuvant CAPOX in II/III rectal cancer patients with low/intermediate risks: study protocol for a prospective, non-inferior, randomized control trial (COPEC trial)
Source: Trials. 2023 Jun 13;24:397. doi: 10.1186/s13063-023-07405-x (PMC10262432; doi:10.1186/s13063-023-07405-x)
Supplement: Supplementary file 2 — Additional file 2: Supplementary Table 1. Criteria of the magnetic resonance tumor regression grade (mrTRG). [file 13063_2023_7405_MOESM2_ESM.docx]

Supplementary Table 1 Criteria of the magnetic resonance tumor regression grade (mrTRG).

| Grades | Extent Description |
| --- | --- |
| mrTRG 1 | Complete radiological response (linear scar only) |
| mrTRG 2 | Good response (dense fibrosis, no obvious tumor signal) |
| mrTRG 3 | Moderate response (>50% fibrosis and visible intermediate signal) |
| mrTRG 4 | Slight response (mostly tumor) |
| mrTRG 5 | No response/re-growth of tumor |
