# Supplementary material for: Comparison of the pathological response to 2 or 4 cycles of neoadjuvant CAPOX in II/III rectal cancer patients with low/intermediate risks: study protocol for a prospective, non-inferior, randomized control trial (COPEC trial)
Source: Trials. 2023 Jun 13;24:397. doi: 10.1186/s13063-023-07405-x (PMC10262432; doi:10.1186/s13063-023-07405-x)
Supplement: Supplementary file 3 — Additional file 3: Supplementary Table 2. Criteria of the pathological tumor regression grade (pTRG). [file 13063_2023_7405_MOESM3_ESM.docx]

Supplementary Table 2 Criteria of the pathological tumor regression grade (pTRG).

| Grades | Grades of regression | Extent Description |
| --- | --- | --- |
| pTRG 0 | Total tumor regression | No residual tumor |
| pTRG 1 | Medium tumor regression | Individual/small foci of residual tumor cells |
| pTRG 2 | Mild tumor regression | Residual tumor, massive fibrotic stroma and fibrotic component ≥ 50 % |
| pTRG 3 | No tumor regression | Extensive residual tumor, no or little necrosis of tumor cells, fibrosis component < 50 % |
