# Supplementary material for: Comparison of the pathological response to 2 or 4 cycles of neoadjuvant CAPOX in II/III rectal cancer patients with low/intermediate risks: study protocol for a prospective, non-inferior, randomized control trial (COPEC trial)
Source: Trials. 2023 Jun 13;24:397. doi: 10.1186/s13063-023-07405-x (PMC10262432; doi:10.1186/s13063-023-07405-x)
Supplement: Supplementary file 4 — Additional file 4. Case Report Form (CRF). [file 13063_2023_7405_MOESM4_ESM.docx]

**Comparison of the pathological effect between 2 and 4 cycles neoadjuvant CAPOX for low/intermediate risk II/III rectal cancer:**

**a prospective, non-inferior, randomized, controlled trial**

**(COPEC trial)**

**Case Report Form**

**(CRF)**

**Subject name:**

**Subject initial:**

**Research center:**

**Research center number:**

**Principal investigator:**

**Enrollment time:**

**Random number:**

**Random grouping: □** **2 cycles group □ 4 cycles group**

**Version date:**

Contents

**Direction for the application form ....................................................................................................... 2**

**Patient contact information .................................................................................................................. 3**

**Inclusion and exclusion criteria .........................****.................................................................................. 4**

**Withdrawal criteria .....................................****.......................................................................................... 6**

**Patient basic information .................................................................................................................... 7**

**First admission .........................................................................****.............................................................. 8**

Random group information

Pretreatment tumor assessment

Clinical symptom assessment

Chemotherapy information

**Second admission ................................................................................................................................. 12**

Clinical symptom assessment

Chemotherapy information

**Third admission ................................................................................................................................... 14**

Postchemotherapy tumor assessment

Clinical symptom assessment

Chemotherapy information

**Fourth admission ................................................................................................................................. 17**

Clinical symptom assessment

Chemotherapy information

**Surgery admission ............................................................................................................................... 19**

Clinical symptom assessment

Chemotherapy completion form

Preoperative tumor assessment

Operation information

Postoperative recovery

**Postoperative pathology ....................................................................................................................... 24**

**Postoperative adjuvant radiotherapy and chemotherapy records .................................................. 25**

**Postoperative follow-up form …………………….............................................................................. 26**

**Long-term endpoints ............................................................................................................................ 34**

**Direction for the application form**

**Before filling out the form, please read the following instructions carefully:**

1. The information of qualified participants should be recorded in the CRF.

2. The case report form should be filled in forcefully with a signature pen.

3. The record must be clear and accurate. Errors should be corrected with a center horizontal line, associated with the initials of modifier and modification time.

E.g. ~~58.6~~ 56.8 LGW 00 02 12.

4. “√” should be marked in the box after all the selected items, e.g. 🗹. All columns in the form should be filled with corresponding words or numbers, and must not be left blank.

5. NK should be recorded when the drug dose and using time are unclear.; all inspection items are not checked or missed for some reason, please fill in ND.

6. During the whole study, adverse events should be recorded timely and truthfully, which includes the occurrence time, severity, during time, measures, outcomes. If there are serious adverse events such as disability, death, the mainly responsible units should be informed immediately.

7. Adverse events should be classified by the NCI-CTCAE v5.0.

| **Patient contact information** | |
| --- | --- |
| **Name:** | **Medical record number:** |
| **Research center:** | **ID number:** |
| **Home address**: | |
| **Contact Person 1**  **Relationship**  **Tel.** | |
| **Contact Person 2** **Relationship**  **Tel.** | |
| **Contact Person 3** **Relationship**  **Tel.** | |

| **Inclusion criteria** | | |
| --- | --- | --- |
| 1. Patients aged between 18 to 75 in either sex. | □YES | □NO |
| 2. Patients with low- and intermediate-risk stage II/III rectal cancer evaluated by MRI and TRUS: middle and low rectal cancer (below the peritoneal reflex line): T3a-bN0-1M0, EMVI (±), MRF (-) (≥ 2mm), and middle and high rectal cancer (above the peritoneal turning line): T3a-c/T4aN0-1M0, EMVI (±), MRF (-) (≥2mm); Mesangial lymph node with short diameter ≥8mm or highly suspected metastasis not more than 3; The short diameter of the lateral lymph node ≤7mm; Patients with very low rectal cancer who match the above criteria and can achieve negative circumferential resection margin under extralevator abdominoperineal excision (ELAPE) surgery will be included in the group. | □YES | □NO |
| 3. Fiberoptic colonoscopy or anal examination, the lower boundary of the lesion is ≤12cm from the anal verge. | □YES | □NO |
| 4. No distant metastasis (including suspected lung nodule metastasis) confirmed by chest and abdomen CT examination; no extra-regional lymph node metastasis (≥10mm). | □YES | □NO |
| 5. Pathologically diagnosed rectal adenocarcinoma. | □YES | □NO |
| 6. Eastern Cooperative Oncology Group (ECOG) score: 0-1. | □YES | □NO |
| 7. Patients with primary rectal cancer who have not received surgery (except palliative ostomy), radiotherapy, systemic chemotherapy, or other anti-tumor treatments before enrollment. | □YES | □NO |
| 8. The main organ function is normal, and meets the following standards:  ① Blood routine criteria: HB ≥9g/dL, WBC ≥ 3.5/4.0×10^9^/L, neutrophil ≥ 1.5×10^9^/L, PLT≥ 100×10^9^/L;  ② Blood biochemical criteria: Crea and BIL ≤ 1.0 times the upper limit of normal value (ULN), ALT and AST≤ 2.5 times the upper limit of normal value (ULN), alkaline phosphatase (ALP) ≤ 2.5×UNL, total bilirubin (Tbil) ≤1.5×UNL. | □YES | □NO |
| 9. No history of 5-Fu and platinum drug allergy. | □YES | □NO |
| 10. Females of childbearing age must undergo a pregnancy test (serum or urine) 7 days before enrollment with the negative result, and are willing to use appropriate contraceptive methods during the trial and 8 weeks after the last administration. | □YES | □NO |
| 11. Patients voluntarily joining the study and signing informed consent forms with good compliance and follow-up. | □YES | □NO |
| **Remarks: All of the above criteria must be "YES", otherwise the patient cannot be enrolled.** | | |

| **Exclusion criteria** | | |
| --- | --- | --- |
| 1. Patients considering lynch syndrome. | □YES | □NO |
| 2. Patients not considering metastasis in the initial diagnosis, but proved to be distant metastases during the treatment | □YES | □NO |
| 3. Previously or concurrently suffering from other malignant tumors (including concurrent colon cancer), except for cured skin basal cell carcinoma and cervical carcinoma in situ | □YES | □NO |
| 4. Pregnant or nursing women. | □YES | □NO |
| 5. Lateral lymph nodes ≥7mm. | □YES | □NO |
| 6. Patients with severe cardiovascular disease and diabetes that are difficult to control. | □YES | □NO |
| 7. Patients with mental disorders. | □YES | □NO |
| 8. Patients with severe infection. | □YES | □NO |
| 9. Patients undergoing thrombolysis/anticoagulation therapy with bleeding diathesis or coagulation dysfunction, or suffering aneurysm, stroke, transient ischemic attack, arteriovenous malformation occurred in the past year. | □YES | □NO |
| 10. Patients with a history of kidney disease, urinary protein, or clinically abnormal renal function. | □YES | □NO |
| 11. Patients with a history of gastrointestinal fistula, perforation, bleeding, or severe ulcer. | □YES | □NO |
| 12. Patients with severe gastrointestinal diseases that affect the absorption of oral chemotherapy drugs. | □YES | □NO |
| 13. Patients participating in another clinical trial within 4 weeks before treatment. | □YES | □NO |
| 14. Patients pathologically diagnosed with mucinous component or the signet-ring cell carcinoma. | □YES | □NO |
| **Remarks: All of the above criteria must be "NO", otherwise the patient cannot be enrolled.** | | |

| **Withdrawal criteria** | | |
| --- | --- | --- |
| 1. Patients refusing further treatment. | □YES | □NO |
| 2. Patients having severe adverse reactions to chemotherapy and being unable to complete 2 cycles of chemotherapy. | □YES | □NO |
| **Remarks: All of the above criteria must be "NO", otherwise the patient cannot be enrolled.** | | |

**Informed consent signed time (month/day/year)：**

**Doctor's declaration (doctor-declare)：**

I have carefully checked the major medical history, inclusion and exclusion criteria. I considered the patient who met the inclusion criteria could be allowed to participate in the study.

| **Patient basic information** | | | | | | |
| --- | --- | --- | --- | --- | --- | --- |
| **Sex:** □M □F | **Age:**  y | **Height:** cm | | | **Weight:** kg | **BMI**： kg/m^2^  BSA： m^2^ |
| **PS score：** | **ASA score ：** | **Complication：**□No □Yes (please give details) | | | | |
| **Family history of cancer** ：□No □ 1 family member □ 2 family members □3 or more family members □ FAP family □ Lynch syndrome | | | | | | |
| **Lab Examination at presentation** | | | | **Inspection date(month/day/year):** | | |
| Hb： g/L；WBC： *10^9^/L；PLT： *10^9^/L； Neutrophil *10^9^/L ；  CEA： ng/mL；CA19-9： ng/mL | | | | | | |
| **Colonoscopy report** | | | | **Inspection date (month/day/year):** | | |
| Distance From anal verge: cm | Circumferential growth: cycle | | Colonoscopy up to the cecum?  □Yes □No | | Pathology of Biopsy： | |
| Colon merger: □no □single polyp □multiple polyps □simultaneous colon cancer (should be excluded) | | | | | | |
| **Electrocardiograph** | | **Inspection date (month/day/year)：** | | | | |
| **Results**:□normal □abnormal □ not checked | | (If “abnormal” please give details ) | | | | |
| **Pulmonary function test** | | **Inspection date (month/day/year)：** | | | | |
| **Results**:□normal □abnormal □ not checked | | ( If “abnormal” please give details ) | | | | |
| **Ultrasonic cardiogram** | | **Inspection date (month/day/year)：** | | | | |
| **Results**:□normal □abnormal □ not checked | | ( If “abnormal” please give details ) | | | | |
| **ECOG score：**□ 0 □1 point □2 points □3 points □4 points □5 points | | | | | | |

（Note: ECOG score: 0, the acting ability is completely normal; 1 point, patients can walk freely and engage in light physical work, but cannot engage in heavy physical work; 2 points, patients can walk freely and take care of themselves but have lost the ability to work, and patients can get up and move with no less than half of the day; 3 points, patients can only partially take care of life, and require on the bed or in the wheelchair for more than half of the day; 4 points, patients are bedridden and unable to take care of life; 5 points, death.）

**First admission**

**Whether to enter random：□Yes □No；**

**Reasons for not entering：□Withdrawal of informed consent;**

**□Toxic and side effects;**

**□Others:**

**Randomization：**

**Random date (month/day/year): ________________________________**

**Random number: ____________________________________________**

**Random grouping****: □2 cycles group □4 cycles group**

| **Pretreatment tumor assessment** | |
| --- | --- |
| **Transrectal ultrasound （**□Yes □No**）** | **Imaging Date****(month/day/year)：** |
| **T ;** Invading the intestinal wall mm; distance from the circumference mm  Tumor longitudinal length : mm ; Tumor width: mm | |
| **Abdominal CT report (Required)（**□no □plain □enhanced**）：**  **Metastases: ○Liver ○Peritoneum (Imaging PCI Score) ______ ○Others __________**  **Imaging Date(month/day/year)：** | |
| **Upper abdominal MRI report（**□Yes □No**）：**  **Imaging Date(month/day/year)：** | |
| **Pelvic MRI (Required)（**□Yes □No**）** | **Imaging Date(month/day/year)：** |
| **T**  Distance from anal verge cm  Tumor longitudinal length mm  T stage  Invading the intestinal wall mm  □ MRF (+)  Distance from the circumference mm  Arc of circumferential involvement %  EMVI score：_____  Tumor growth pattern: □annular □polypoid □mucous □ulceration □perforation  Deepest invasive location: □anterior □posterior □right side □left side | **N**  the maximum diameter of the largest LN mm  LN Number  Numbers of ≥10mm LN  Suspicious LNs outside the mesentery: □ Abdominal-aorta, mediastinum and neck; □Left-internal iliac (≥5mm); □Left-obturator; □Right-internal iliac; □Right-obturator; □Left inguinal area; □Right inguinal area; □No |
| **Chest CT report（**□Yes □No**）：**  **Imaging Date(month/day/year)：** | |
| **Physical examination（**□Yes □No **）** | **Distance by DRE cm；Others：** |
| **Colonoscopy（**□Yes □No **）** | **Distance by colonoscopy cm； Others：** |
| **Liver contrast-enhanced ultrasound report（**□Yes □No **）：**  **Imaging Date(month/day/year)：** | |
| **Site of metastasis:** □No □Liver □Lung □Bone □Peritoneal □Ovary □Brain □Extra-Regional LNs □Others | |
| **cTNM stage: cT N M** | |
| **Note:** | |

**Clinical symptom assessment before treatment**

| **In the past two weeks,**  **have you had any of the following symptoms?** | None | Occasionally | Often | Severe/Always |
| --- | --- | --- | --- | --- |
| 1. constipation and difficulty in defecation | ○ | ○ | ○ | ○ |
| 2. diarrhea | ○ | ○ | ○ | ○ |
| 3. blood in your stools | ○ | ○ | ○ | ○ |
| 4. mucus in your stools | ○ | ○ | ○ | ○ |
| 5. thin stools | ○ | ○ | ○ | ○ |
| 6. abdominal pain | ○ | ○ | ○ | ○ |
| 7. lower back pain | ○ | ○ | ○ | ○ |
| 8. bulging anus and having incessant bowel movements | ○ | ○ | ○ | ○ |

| **First cycle of chemotherapy** | | | | |
| --- | --- | --- | --- | --- |
| **Regimen** | **Capecitabine Oxaliplatin** | | | Start date(month/day/year):  End date(month/day/year): |
| **Chemotherapeutic side effects （□No □Yes）** | | | | |
| □ Hb | | Diagnostic basis： | Severity grade：□ 1° □2° □3° □4° | |
| □ WBC | | Diagnostic basis： | Severity grade：□ 1° □2° □3° □4° | |
| □ Neutrophil | | Diagnostic basis： | Severity grade：□ 1° □2° □3° □4° | |
| □ PLT | | Diagnostic basis： | Severity grade：□ 1° □2° □3° □4° | |
| □ Liver damage | | Diagnostic basis： | Severity grade：□ 1° □2° □3° □4° | |
| □ Kidney damage | | Diagnostic basis： | Severity grade：□ 1° □2° □3° □4° | |
| □ Vomit | | Diagnostic basis： | Severity grade：□ 1° □2° □3° □4° | |
| □ Diarrhea | | Diagnostic basis： | Severity grade：□ 1° □2° □3° □4° | |
| □ Constipation | | Diagnostic basis： | Severity grade：□ 1° □2° □3° □4° | |
| □ Cardiotoxicity | | Diagnostic basis： | Severity grade：□ 1° □2° □3° □4° | |
| □ Allergic reaction | | Diagnostic basis： | Severity grade：□ 1° □2° □3° □4° | |
| □ Hand-foot skin reaction | | Diagnostic basis： | Severity grade：□ 1° □2° □3° □4° | |
| □ Sensory neuropathy | | Diagnostic basis： | Severity grade：□ 1° □2° □3° □4° | |
| □ Others | | Diagnostic basis： | | |
| **Other medication conditions：** | |  | | |
| **Is the course of treatment delayed? □Yes □No** | | | | |

**Second admission**

**Clinical symptom assessment after 1 cycle of chemotherapy**

| **In the past two weeks,**  **have you had any of the following symptoms?** | None | Lighter than the previous cycle | Similar to or worse than the previous cycle |
| --- | --- | --- | --- |
| 1. constipation and difficulty in defecation | ○ | ○ | ○ |
| 2. diarrhea | ○ | ○ | ○ |
| 3. blood in your stools | ○ | ○ | ○ |
| 4. mucus in your stools | ○ | ○ | ○ |
| 5. thin stools | ○ | ○ | ○ |
| 6. abdominal pain | ○ | ○ | ○ |
| 7. lower back pain | ○ | ○ | ○ |
| 8. bulging anus and having incessant bowel movements | ○ | ○ | ○ |

| **Second cycle of chemotherapy** | | | | | |
| --- | --- | --- | --- | --- | --- |
| **Regimen** | **Capecitabine Oxaliplatin** | | | | Start date(month/day/year):  End date(month/day/year): |
| **Whether to reduce the dose of chemotherapy** | Reasons for reduction and adjustment： | | **Whether to delay the start of chemotherapy** | | Reason for delay： |
| **Chemotherapeutic side effects （□No □Yes）** | | | | | |
| □ Hb | | Diagnostic basis： | | Severity grade：□ 1° □2° □3° □4° | |
| □ WBC | | Diagnostic basis： | | Severity grade：□ 1° □2° □3° □4° | |
| □ Neutrophil | | Diagnostic basis： | | Severity grade：□ 1° □2° □3° □4° | |
| □ PLT | | Diagnostic basis： | | Severity grade：□ 1° □2° □3° □4° | |
| □ Liver damage | | Diagnostic basis： | | Severity grade：□ 1° □2° □3° □4° | |
| □ Kidney damage | | Diagnostic basis： | | Severity grade：□ 1° □2° □3° □4° | |
| □ Vomit | | Diagnostic basis： | | Severity grade：□ 1° □2° □3° □4° | |
| □ Diarrhea | | Diagnostic basis： | | Severity grade：□ 1° □2° □3° □4° | |
| □ Constipation | | Diagnostic basis： | | Severity grade：□ 1° □2° □3° □4° | |
| □ Cardiotoxicity | | Diagnostic basis： | | Severity grade：□ 1° □2° □3° □4° | |
| □ Allergic reaction | | Diagnostic basis： | | Severity grade：□ 1° □2° □3° □4° | |
| □ Hand-foot skin reaction | | Diagnostic basis： | | Severity grade：□ 1° □2° □3° □4° | |
| □ Sensory neuropathy | | Diagnostic basis： | | Severity grade：□ 1° □2° □3° □4° | |
| □ Others | | Diagnostic basis： | | | |
| **Other medication conditions：** | |  | | | |
| **Is the course of treatment delayed? □Yes □No** | | | | | |

**Third admission**

**Clinical symptom assessment after 2 cycles of chemotherapy**

| **In the past two weeks,**  **have you had any of the following symptoms?** | None | Lighter than the previous cycle | Similar to or worse than the previous cycle |
| --- | --- | --- | --- |
| 1. constipation and difficulty in defecation | ○ | ○ | ○ |
| 2. diarrhea | ○ | ○ | ○ |
| 3. blood in your stools | ○ | ○ | ○ |
| 4. mucus in your stools | ○ | ○ | ○ |
| 5. thin stools | ○ | ○ | ○ |
| 6. abdominal pain | ○ | ○ | ○ |
| 7. lower back pain | ○ | ○ | ○ |
| 8. bulging anus and having incessant bowel movements | ○ | ○ | ○ |

| **Tumor assessment** **after 2 cycles of chemotherapy** | |
| --- | --- |
| Weight：_________Kg | **BMI**： kg/m^2^  BSA： m^2^ |
| **3-4 level of Side effects in the first 2 cycles:**  **□No □** **Yes, but no need to reduce □** **Yes, chemotherapy needs to be stopped or dose reduced** | |
| **Transrectal ultrasound （**□Yes □No**）** | **Imaging Date(month/day/year)：** |
| **T** ; Invading the intestinal wall mm; distance from the circumference mm  Tumor longitudinal length : mm ; Tumor width: mm | |
| **Abdominal CT report (Required)（**□no □plain □enhanced**）：**  **Metastases: ○Liver ○Peritoneum (Imaging PCI Score) ______ ○Others __________**  **Imaging Date(month/day/year)：** | |
| **Pelvic MRI (Required)（**□Yes □No**）** | **Imaging Date(month/day/year)：** |
| **T**  Distance from anal verge cm  Tumor longitudinal length mm  T stage  Invading the intestinal wall mm  □ MRF (+)  Distance from the circumference mm  Arc of circumferential involvement %  EMVI score：_____  Tumor growth pattern: □annular □polypoid □mucous □ulceration □perforation  Deepest invasive location: □anterior □posterior □right side □left side  □ Residual tumor (T2-weighted hyperintensity)  □ Fibrosis (low signal on T2 weight)  mTRG: □1 □2 □3 □4 □5  mRECIST (based on tumor longitudinal diameter): □CR □PR □SD □PD (10%) | **N**  The maximum diameter of the largest LN mm  LN Number  Numbers of ≥10mm LN  Suspicious LNs outside the mesentery:  □ Abdominal-aorta, mediastinum and neck; □Left-internal iliac (≥5mm); □Left-obturator; □Right-internal iliac; □Right-obturator; □Left inguinal area; □Right inguinal area; □ No  **The reaction of the mesangial LNs**:  □ Disappeared; □ All shrunk significantly; □ No significant change; □ Individual increased; □ Multiple LN increased  **The reaction of the extraregional LNs**:  □ Significant shrinkage or disappearance; □ No significant change; □ Significant increase □ Confirmed metastasis (should be withdrawn) |
| **Physical examination（**□Yes □No **）** | **Distance by DRE cm；Others：** |
| **Tumor size compared with 0 cycle:** □ Disappeared or only scar remained □ Significantly reduced □ No obvious change □ Increased □ Unpalpable | |
| **Site of metastasis:** □No □Liver □Lung □Bone □Peritoneal □Ovary □Brain □Extra-Regional LNs □Others | |
| **Stage: 2-ycT N M** | |
| **Note:** | |

| **Third cycle of chemotherapy** | | | | | |
| --- | --- | --- | --- | --- | --- |
| **Regimen** | **Capecitabine Oxaliplatin** | | | | Start date(month/day/year):  End date(month/day/year): |
| **Whether to reduce the dose of chemotherapy** | Reasons for reduction and adjustment： | | **Whether to delay the start of chemotherapy** | | Reason for delay： |
| **Chemotherapeutic side effects （□No □Yes）** | | | | | |
| □ Hb | | Diagnostic basis： | | Severity grade：□ 1° □2° □3° □4° | |
| □ WBC | | Diagnostic basis： | | Severity grade：□ 1° □2° □3° □4° | |
| □ Neutrophil | | Diagnostic basis： | | Severity grade：□ 1° □2° □3° □4° | |
| □ PLT | | Diagnostic basis： | | Severity grade：□ 1° □2° □3° □4° | |
| □ Liver damage | | Diagnostic basis： | | Severity grade：□ 1° □2° □3° □4° | |
| □ Kidney damage | | Diagnostic basis： | | Severity grade：□ 1° □2° □3° □4° | |
| □ Vomit | | Diagnostic basis： | | Severity grade：□ 1° □2° □3° □4° | |
| □ Diarrhea | | Diagnostic basis： | | Severity grade：□ 1° □2° □3° □4° | |
| □ Constipation | | Diagnostic basis： | | Severity grade：□ 1° □2° □3° □4° | |
| □ Cardiotoxicity | | Diagnostic basis： | | Severity grade：□ 1° □2° □3° □4° | |
| □ Allergic reaction | | Diagnostic basis： | | Severity grade：□ 1° □2° □3° □4° | |
| □ Hand-foot skin reaction | | Diagnostic basis： | | Severity grade：□ 1° □2° □3° □4° | |
| □ Sensory neuropathy | | Diagnostic basis： | | Severity grade：□ 1° □2° □3° □4° | |
| □ Others | | Diagnostic basis： | | | |
| **Other medication conditions：** | | **Other medication conditions：** | | | |
| **Is the course of treatment delayed? □Yes □No** | | | | | |

**Fourth admission**

**Clinical symptom assessment after 3 cycles of chemotherapy**

| **In the past two weeks,**  **have you had any of the following symptoms?** | None | Lighter than the previous cycle | Similar to or worse than the previous cycle |
| --- | --- | --- | --- |
| 1. constipation and difficulty in defecation | ○ | ○ | ○ |
| 2. diarrhea | ○ | ○ | ○ |
| 3. blood in your stools | ○ | ○ | ○ |
| 4. mucus in your stools | ○ | ○ | ○ |
| 5. thin stools | ○ | ○ | ○ |
| 6. abdominal pain | ○ | ○ | ○ |
| 7. lower back pain | ○ | ○ | ○ |
| 8. bulging anus and having incessant bowel movements | ○ | ○ | ○ |

| **Fourth cycle of chemotherapy** | | | | | |
| --- | --- | --- | --- | --- | --- |
| **Regimen** | **Capecitabine Oxaliplatin** | | | | Start date(month/day/year):  End date(month/day/year): |
| **Whether to reduce the dose of chemotherapy** | Reasons for reduction and adjustment： | | **Whether to delay the start of chemotherapy** | | Reason for delay： |
| **Chemotherapeutic side effects （□No □Yes）** | | | | | |
| □ Hb | | Diagnostic basis： | | Severity grade：□ 1° □2° □3° □4° | |
| □ WBC | | Diagnostic basis： | | Severity grade：□ 1° □2° □3° □4° | |
| □ Neutrophil | | Diagnostic basis： | | Severity grade：□ 1° □2° □3° □4° | |
| □ PLT | | Diagnostic basis： | | Severity grade：□ 1° □2° □3° □4° | |
| □ Liver damage | | Diagnostic basis： | | Severity grade：□ 1° □2° □3° □4° | |
| □ Kidney damage | | Diagnostic basis： | | Severity grade：□ 1° □2° □3° □4° | |
| □ Vomit | | Diagnostic basis： | | Severity grade：□ 1° □2° □3° □4° | |
| □ Diarrhea | | Diagnostic basis： | | Severity grade：□ 1° □2° □3° □4° | |
| □ Constipation | | Diagnostic basis： | | Severity grade：□ 1° □2° □3° □4° | |
| □ Cardiotoxicity | | Diagnostic basis： | | Severity grade：□ 1° □2° □3° □4° | |
| □ Allergic reaction | | Diagnostic basis： | | Severity grade：□ 1° □2° □3° □4° | |
| □ Hand-foot skin reaction | | Diagnostic basis： | | Severity grade：□ 1° □2° □3° □4° | |
| □ Sensory neuropathy | | Diagnostic basis： | | Severity grade：□ 1° □2° □3° □4° | |
| □ Others | | Diagnostic basis： | | | |
| **Other medication conditions：** | | **Other medication conditions：** | | | |
| **Is the course of treatment delayed? □Yes □No** | | | | | |

**Surgery admission**

**Clinical symptom assessment after 4 cycles of chemotherapy**

| **In the past two weeks,**  **have you had any of the following symptoms?** | None | Lighter than the previous cycle | Similar to or worse than the previous cycle |
| --- | --- | --- | --- |
| 1. constipation and difficulty in defecation | ○ | ○ | ○ |
| 2. diarrhea | ○ | ○ | ○ |
| 3. blood in your stools | ○ | ○ | ○ |
| 4. mucus in your stools | ○ | ○ | ○ |
| 5. thin stools | ○ | ○ | ○ |
| 6. abdominal pain | ○ | ○ | ○ |
| 7. lower back pain | ○ | ○ | ○ |
| 8. bulging anus and having incessant bowel movements | ○ | ○ | ○ |

| **Chemotherapy completion form** | | | |
| --- | --- | --- | --- |
| **Initial chemotherapy regimen：**Capecitabine Oxaliplatin | | | |
| Start date(month/day/year): | | End date(month/day/year): | Number of cycles: cycles |
| Is it completed normally:  □Yes □No | | Whether to delay: □Yes □No | Whether to change the regimen: □Yes □No |
| **Total dose of chemotherapy** | | Capecitabine Oxaliplatin | |
| **Dose adjustment records** | | | |
| Date(month/day/year): | | Capecitabine Oxaliplatin | Reason for adjustment： |
| Date(month/day/year): | | Capecitabine Oxaliplatin | Reason for adjustment： |
| Date(month/day/year): | | Capecitabine Oxaliplatin | Reason for adjustment： |
| **Chemotherapeutic side effects (**□No □Yes**)** | | | |
| No.1 | □Hemoglobin reduction □leukopenia □granulocytopenia □ diarrhea □constipation □cardiotoxicity □allergic reaction □hand-foot skin reaction □sensory neuropathy □others _______ | | Severity grade：  □ 1° □2° □3° □4° |
| No.2 | □Hemoglobin reduction □leukopenia □granulocytopenia □ diarrhea □constipation □cardiotoxicity □allergic reaction □hand-foot skin reaction □sensory neuropathy □others _______ | | Severity grade：  □ 1° □2° □3° □4° |
| No.3 | □Hemoglobin reduction □leukopenia □granulocytopenia □ diarrhea □constipation □cardiotoxicity □allergic reaction □hand-foot skin reaction □sensory neuropathy □others _______ | | Severity grade：  □ 1° □2° □3° □4° |
| No.4 | □Hemoglobin reduction □leukopenia □granulocytopenia □ diarrhea □constipation □cardiotoxicity □allergic reaction □hand-foot skin reaction □sensory neuropathy □others _______ | | Severity grade：  □ 1° □2° □3° □4° |

| **Preoperative tumor assessment** | |
| --- | --- |
| Weight：_________Kg | **BMI**： kg/m^2^  BSA： m^2^ |
| **Transrectal ultrasound （**□Yes □No**）** | **Imaging Date(month/day/year)：** |
| **T** ; Invading the intestinal wall mm; distance from the circumference mm  Tumor longitudinal length : mm ; Tumor width: mm | |
| **Abdominal CT report (Required)（**□no □plain □enhanced**）：**  **Metastases: ○Liver ○Peritoneum (Imaging PCI Score) ______ ○Others __________**  **Imaging Date(month/day/year)：** | |
| **Pelvic MRI (Required)（**□Yes □No**）** | **Imaging Date(month/day/year)：** |
| **T**  Distance from anal verge cm  Tumor longitudinal length mm  T stage  Invading the intestinal wall mm  □ MRF (+)  Distance from the circumference mm  Arc of circumferential involvement %  EMVI score：_____  Tumor growth pattern: □annular □polypoid □mucous □ulceration □perforation  Deepest invasive location: □anterior □posterior □right side □left side  □ Residual tumor (T2-weighted hyperintensity)  □ Fibrosis (low signal on T2 weight)  mTRG: □1 □2 □3 □4 □5  mRECIST (based on tumor longitudinal diameter): □CR □PR □SD □PD (10%) | **N**  The maximum diameter of the largest LN mm  LN Number  Numbers of ≥10mm LN  Suspicious LNs outside the mesentery:  □ Abdominal-aorta, mediastinum and neck; □Left-internal iliac (≥5mm); □Left-obturator; □Right-internal iliac; □Right-obturator; □Left inguinal area; □Right inguinal area; □ No  **The reaction of the mesangial LNs**:  □ Disappeared; □ All shrunk significantly; □ No significant change; □ Individual increased; □ Multiple LN increased  **The reaction of the extraregional LNs**:  □ Significant shrinkage or disappearance; □ No significant change; □ Significant increase □ Confirmed metastasis (should be withdrawn) |
| **Physical examination（**□Yes □No**）**  **Distance by DRE**  **cm** | **Inspection date (month/day/year):** |
| **Tumor size compared with 0 cycle:** □ Disappeared or only scar remained □ Significantly reduced □ No obvious change □ Increased □ Unpalpable | |
| **Colonoscopy results:** Distance from anal verge cm;  Efficacy evaluation: □Disappeared or only residual fibrotic scars; □ Significant retraction but still a raised neoplasm or ulcer; □ Unchanged; □ Increased | |
| **Site of metastasis:** □No □Liver □Lung □Bone □Peritoneal □Ovary □Brain □Extra-Regional LNs □Others | |
| **ycTNM stage** | |
| Hb： g/L；WBC： *10^9^/L；PLT： *10^9^/L； Neutrophil *10^9^/L ；  CEA： ng/mL；CA19-9： ng/mL； | |

**Operation information**

| **Preoperative complications** (please give details): | | |
| --- | --- | --- |
| **Performing operation**: □Yes □No；  Cause of no operation: □cCR or near cCR □Local or distant progression □Patient withdrawal □others | | |
| **Operation date**(month/day/year)：_______________________ | | **Interval between surgery and radiotherapy**：_________ (d) |
| **ASA score：** | **Surgeon's name：** | |
| **DVT Caprini score at initial diagnosis：**□Very low risk = 0 □Low risk 1-2 points □medium risk 3-4 points □High risk ≥5 points  ECOG score：______ | | |

**Intraoperative**

| **Type of surgery:**  □Selective surgery □Emergency surgery | | | **Rectal cancer status:** □ No special □ Incomplete ileus □ Acute ileus □ Perforation □ Intestinal obstruction after stent placement □ Major bleeding □ No obstruction but poor bowel preparation | | | | | | | | | |
| --- | --- | --- | --- | --- | --- | --- | --- | --- | --- | --- | --- | --- |
| **Tumor location:**  □Reflexion upper □Reflexion □Reflexion lower □Anal canal | | | | | Circumferential growth: □≤1/4 □1/3 □1/2 □3/4 □Full circumference | | | | | | | |
|  |  |  |  |  | **Tumor size:** × cm | | | | **Whether the anterior wall of the rectum is involved:** □Yes □No | | | |
| **The deepest tumor is located in:** □ Front □ Rear □ Left side □ Left front □ Left rear □ Right side □ Right front □ Right rear | | | | | | | | | **Gross classification:** □mass □ulcer type □peripheral infiltrating type □combined with intra-mesangial perforation | | | |
| **Tumor perforation:**  □Yes □No | **Invasion of periintestinal tissues and organs**: □Yes □No | | | | | **Invaded tissues and organs**: | | | | | | |
| **Distant metastasis**：□No □Yes | | | | **Metastasis site:** □ Peritoneal (PCI): □ Liver□ Lateral lymph node □ Retroperitoneal | | | | | | | | |
| **Curability intention**：  □curative surgery □Palliative surgery  □non-resectable | | | | **Surgery type:**  □Laparotomy □Laparoscopic surgery(include handassisted)□robotic □Laparoscopic surgery converted to laparotomy | | | | | | **Reason of conversion**： | | |
| **Surgical procedures:**  □ MILES □ELAPE □Dixon □Dixon drags out DST □ Hartmann □ Transanal local resection □ISR or Parks □NOSES □TaTME □Others | | | | | | | | | | | | |
| **Left colic artery preserved:** □Yes □No | | | | | | | | | | | | |
| **Lymph nodes dissection：**  □D1 □D2 □D3 | | | | **Lateral lymph node dissection:**  □No □Left □Right □Bilateral | | | | | | **Excessive LN resection**： | | |
| **Anastomosis method:**  □End-to-side □ End-to-end □ J-pouch | | | | **Prophylactic stoma:** □Yes □No | | | **Anastomosis method**：  □Colo-anal □Colo-rectal | | | **Coloanal anastomosis method**:  □Manual □Apparatus | | |
| **Stapler size**：  □28 or 29mm □31-33mm | | **The distance from anastomosis to anal verge**: cm | | | | | **Anus placement**：  □Yes □No | | | **CRM prediction:**  □Positive □Indeterminate □Negative | | |
| **Mesangial integrity self-assessment**:  **Anus-preserving procedure**: □Poor (exposed muscle layer) □Inner mesentery plane □Good (outer mesentery plane)  **Miles procedure**: □A (perforation) □B (intestinal muscle plane) □C (external plane of levator ani) | | | | | | | | | | | | |
| **Degree of resection:**  □R0 □R1 □R2 | | **Residual tumor site**： | | | **Combined organ resection:** □No □Yes Removal of organs: | | | | | | **Reason for excision**： | |
| **Distal margin:** cm | | **Proximal margin**: cm | | |  | | | | | | | |
| **Total operation (min)：** | | **Incision length(cm)**： | | | **Blood loss (ml)**： | | | **Transfusion**：□Yes □No | | | | **The reason for transfusion：**  □Hemorrhage  □Anemia before surgery |
| **Blood transfusion** (ml): Red suspension U | | | | | **Intraoperative intraperitoneal chemotherapy**：  □Yes □No | | | | | | | **Drug name**： |
| **Dosage**： | | **Operative complication**：□Yes □No | | | If yes, please describe： | | | | | | | |

| **Postoperative recovery** | | | | |
| --- | --- | --- | --- | --- |
| **First flatus: ___ (h)** | **Full liquid diet: ___ (h)** | | **Postoperative hospital stay: ___ (d)** | **Healing time ___(d)** |
| **Postoperative complications：**  □Anastomotic leakage □Intra-abdominal Bleeding □Intraluminal bleeding □Bowel Necrosis □Pancreatic fistula □Biliary fistula □Hepatic abscess or infection □Hepatic hematoma □Chylous fistula □Intra-abdominal infection □Inflammatory ileus □Mechanical ileus □Gastrointestinal dysfunction □Subcutaneous emphysema □Incision infection □Wound disruption □Incisional hernia □Pulmonary infection □Urinary infection □DVT □Pulmonary embolism □Cardiac complication □Complication in CNS □Uroschesis □Others | | | | |
| **Clavien system grading：**□**I** □**II** □**IIIa** □**IIIb** □**IVa** □**IVb** □**V** | | | | |
| **Postoperative transfusion ：**□**Yes** □**No** | |  | | |
| **Perioperative death：**□**Yes** □**No** | | **Causes of death:** | | |
| **Reoperation:** □**Yes** □**No** | | **Causes of reoperation:** | | |
| **Unplanned readmission within 30 days**: □**Yes** □**No** | | **Causes of readmission**： | | |

**Postoperative pathology**

| **Histological classification：**□Adenocarcinoma □Others | | | | | | | |
| --- | --- | --- | --- | --- | --- | --- | --- |
| **Tumor size:**  × × cm | **Maximum diameter:**  cm | | **pT** **staging**：□pTis □pT1 □pT2 □pT3 □T4a □T4b | | | | |
| **Grading**: □Gx □G1 □G2 □G3 □G4 | | | **Patho-depth of extramural invasion for T3** ______mm | | | | **P-distance to CRM** _____mm |
| **Total meso-LN evaluated** | **Total Positive meso-LN** | | | **Extraregional positive LN:** □Yes □No | | | |
| **N stage**：  □N0 □N1a □N1b □N1c □N2a □N2b □Nx | | | **Distant metastasis**：  □ Mx □ M0 □ M1a □M1b | | | | **Metastasis site**： |
| **Proximal margin**:  □Negative □Tumor residue | | **Distal incisal margin**:  □Negative □Tumor residue | | | **Mesenteric margin**:  □Negative □Tumor residue | | |
| **Invasion of lymph vessels**：  □Yes □No | | **Invasion of veins**：□Yes □No | | | **Perineural invasion**：□Yes □No | | |
| **Paratumor deposits：**  □Yes □No | | **Tumor budding:**  □Low grade (0-4 buds) □Medium grade (5-9 buds) □High grade (≥10 buds) | | | | | |
| **Pathological mesangial integrity**：  □Poor (muscle layer) □Inner mesentery □Good (mesentery plane) | | | **Pathological Miles Score**：  □A (perforation) □B (intestinal muscle plane) □C (external plane of levator ani) | | | | |
| **pTNM staging**：□0 □1 □2A □2B □2C □3A □3B □3C □4A □4B | | | | | | **CRM**： □ Postive □Negative | |
| **Evaluation of pTRG by sub-centers**：  □TRG0（pCR） □TRG1 (Individual/small foci of residual tumor cells) □TRG2 (Obvious residual tumor and massive fibrotic stroma) □TRG3(No obvious regression) | | | | | | | |
| **Evaluation of pTRG by the Central Pathology Panel：**  □TRG0（pCR） □TRG1 (Individual/small foci of residual tumor cells) □TRG2 (Obvious residual tumor and massive fibrotic stroma) □TRG3(No obvious regression) | | | | | | | |
| □KRAS □NRAS □BRAF □HER2 Ki67 ；MLH1 ；MSH2 ；PMS2 ; MLH6 ; | | | | | | | |

# Postoperative adjuvant radiotherapy and chemotherapy records

**Reasons for postoperative supplemental adjuvant radiotherapy**：

Postoperative supplemental adjuvant radiotherapy total dose： Gy

Start date(month/day/year) ~ End date(month/day/year)

Concurrent medication: □5-FU single drug □None □Others

Radiation dose reduction：□Yes：Total dose Gy；□No

Radiotherapy-related side effects and their grades(Please record the most severe one for the same side effect)：

**Reasons for postoperative supplemental adjuvant chemotherapy:**

Postoperative supplemental adjuvant chemotherapy regimens:

□FOLFOX □XELOX □FOLFIRI □FOLFOXIRI □5FU/LV □Cetuximab □Bevacizumab □Others

Total cycles： cycles

Start date (month/day/year) ~ End date (month/day/year)

Completion status of adjuvant chemotherapy:

□Well □No reduction but delay □Reduction and delay □Early termination

Reasons for changing chemotherapy regimens：

Chemotherapy-related side effects and their grades(Please record the most severe one for the same side effect)：

| **Follow-up chart 1** | **Follow-up date (m/d/y)** | | | | | | **Follow-up methods:**  □Out-patient clinic □Telephone  □Others_____ | | | | | **After operation:**  ____________ months |
| --- | --- | --- | --- | --- | --- | --- | --- | --- | --- | --- | --- | --- |
| **Post-discharge complications**：  □No □Yes（Description and treatments）： | | | | | **Reoperation:** □Yes □No | | | | | **Reason for reoperation**： | | |
| **Outcomes:**  □Death □Alive with tumor □Alive without tumor | | | | | **Death Causes:**  □Cancer specific  □other causes | | | | | **Death Date(m/d/y):** | | |
| **DFS：** | | | | | **OS：** | | | | | **LRFS：** | | |
| **Physical examination:** □Normal □Abnormal  Description of physical examine: | | | | | | | | **Colonoscopy:** □Normal □Abnormal  Report of coloscopy： | | | | |
| **Blood test**：CEA： ng/mL; CA19-9： ng/mL ; WBC： *10^9^/L; Hb： g/L; PLT： *10^9^/L; | | | | | | | | | | | | |
| **Abdominal imaging:**  □ Normal □ Abnormal (please describe): | | | | | | | | **Chest imaging**：  □ Normal □ Abnormal (please describe): | | | | |
| **Abdominal and pelvic ultrasonography：**  □ Normal □ Abnormal (please describe): | | | | | | | | **Ower extremity venous ultrasonography:**  □ Normal □ Abnormal (please describe): | | | | |
| **Metastasis：**  □Yes □No □Suspected | | **Mets diagnosed by：**  □Pathology □imaging  □PET-CT □Physical examination | | | | | | **Date of mets (m/d/y):** | | **Location of mets:**  □ Liver □ Lung □ Bone  □ Retroperitoneal lymph □ Peritoneum □ Brain □ Ovary □Others | | |
| **Resectability**:  □Resectable □convertible □Unresectable | | | | | | **Reoperation for mets**:  □ Yes□ No | | | | **Date of surgery for mets**  **(m/d/y):** | | |
| **Name of Surgery:** | | | | | | **Grading of resection**：  □R0 □R1 □R2 | | | | **Other therapies：**  □None □Chemotherapy  □Radiotherapy □Intervention  □ Radiofrequency □Others | | |
| **Postoperative pathology:** | | | | | | | | | | | | |
| **Local recurrence:**  □Yes □No □Suspected | | **LR diagnosed by：**  □Pathology □imaging  □PET-CT □Physical examination | | | | | | **Date of LR (m/d/y):** | | | **Location of LR**:  □ Anastomosis □ Next to bowel □ Lateral □ Incision | |
| **Rectal recurrence classification:**  □Center type □Forward type  □Side type □Backward type | | | **Recurrence subsides in the lateral region:**  □ Left common iliac □ Left external iliac □ Left internal iliac □ Left obturator  □ Right common iliac □ Right external iliac □ Right internal iliac □ Right obturator | | | | | | | | | |
| **Reoperation for LR:**□ Yes□ No | | | | **Date of surgery for LR**  **(m/d/y):** | | | | | **Name of Surgery:** | | | |
| **Grading of resection**：□R0 □R1 □R2 | | | | **Other therapies：**□None □Chemotherapy □Radiotherapy □Intervention  □ Radiofrequency □Others | | | | | | | | |
| **Other information concerning follow-up：** | | | | | | | | | | | | |

| **Follow-up chart 2** | **Follow-up date (m/d/y)** | | | | | | **Follow-up methods:**  □Out-patient clinic □Telephone  □Others_____ | | | | | **After operation:**  ____________ months |
| --- | --- | --- | --- | --- | --- | --- | --- | --- | --- | --- | --- | --- |
| **Post-discharge complications**：  □No □Yes（Description and treatments）： | | | | | **Reoperation:** □Yes □No | | | | | **Reason for reoperation**： | | |
| **Outcomes:**  □Death □Alive with tumor □Alive without tumor | | | | | **Death Causes:**  □Cancer specific  □other causes | | | | | **Death Date(m/d/y):** | | |
| **DFS：** | | | | | **OS：** | | | | | **LRFS：** | | |
| **Physical examination:** □Normal □Abnormal  Description of physical examine: | | | | | | | | **Colonoscopy:** □Normal □Abnormal  Report of coloscopy： | | | | |
| **Blood test**：CEA： ng/mL; CA19-9： ng/mL ; WBC： *10^9^/L; Hb： g/L; PLT： *10^9^/L; | | | | | | | | | | | | |
| **Abdominal imaging:**  □ Normal □ Abnormal (please describe): | | | | | | | | **Chest imaging**：  □ Normal □ Abnormal (please describe): | | | | |
| **Abdominal and pelvic ultrasonography：**  □ Normal □ Abnormal (please describe): | | | | | | | | **Ower extremity venous ultrasonography:**  □ Normal □ Abnormal (please describe): | | | | |
| **Metastasis：**  □Yes □No □Suspected | | **Mets diagnosed by：**  □Pathology □imaging  □PET-CT □Physical examination | | | | | | **Date of mets (m/d/y):** | | **Location of mets:**  □ Liver □ Lung □ Bone  □ Retroperitoneal lymph □ Peritoneum □ Brain □ Ovary □Others | | |
| **Resectability**:  □Resectable □convertible □Unresectable | | | | | | **Reoperation for mets**:  □ Yes□ No | | | | **Date of surgery for mets**  **(m/d/y):** | | |
| **Name of Surgery:** | | | | | | **Grading of resection**：  □R0 □R1 □R2 | | | | **Other therapies：**  □None □Chemotherapy  □Radiotherapy □Intervention  □ Radiofrequency □Others | | |
| **Postoperative pathology:** | | | | | | | | | | | | |
| **Local recurrence:**  □Yes □No □Suspected | | **LR diagnosed by：**  □Pathology □imaging  □PET-CT □Physical examination | | | | | | **Date of LR (m/d/y):** | | | **Location of LR**:  □ Anastomosis □ Next to bowel □ Lateral □ Incision | |
| **Rectal recurrence classification:**  □Center type □Forward type  □Side type □Backward type | | | **Recurrence subsides in the lateral region:**  □ Left common iliac □ Left external iliac □ Left internal iliac □ Left obturator  □ Right common iliac □ Right external iliac □ Right internal iliac □ Right obturator | | | | | | | | | |
| **Reoperation for LR:**□ Yes□ No | | | | **Date of surgery for LR**  **(m/d/y):** | | | | | **Name of Surgery:** | | | |
| **Grading of resection**：□R0 □R1 □R2 | | | | **Other therapies：**□None □Chemotherapy □Radiotherapy □Intervention  □ Radiofrequency □Others | | | | | | | | |
| **Other information concerning follow-up：** | | | | | | | | | | | | |

| **Follow-up chart 3** | **Follow-up date (m/d/y)** | | | | | | **Follow-up methods:**  □Out-patient clinic □Telephone  □Others_____ | | | | | **After operation:**  ____________ months |
| --- | --- | --- | --- | --- | --- | --- | --- | --- | --- | --- | --- | --- |
| **Post-discharge complications**：  □No □Yes（Description and treatments）： | | | | | **Reoperation:** □Yes □No | | | | | **Reason for reoperation**： | | |
| **Outcomes:**  □Death □Alive with tumor □Alive without tumor | | | | | **Death Causes:**  □Cancer specific  □other causes | | | | | **Death Date(m/d/y):** | | |
| **DFS：** | | | | | **OS：** | | | | | **LRFS：** | | |
| **Physical examination:** □Normal □Abnormal  Description of physical examine: | | | | | | | | **Colonoscopy:** □Normal □Abnormal  Report of coloscopy： | | | | |
| **Blood test**：CEA： ng/mL; CA19-9： ng/mL ; WBC： *10^9^/L; Hb： g/L; PLT： *10^9^/L; | | | | | | | | | | | | |
| **Abdominal imaging:**  □ Normal □ Abnormal (please describe): | | | | | | | | **Chest imaging**：  □ Normal □ Abnormal (please describe): | | | | |
| **Abdominal and pelvic ultrasonography：**  □ Normal □ Abnormal (please describe): | | | | | | | | **Ower extremity venous ultrasonography:**  □ Normal □ Abnormal (please describe): | | | | |
| **Metastasis：**  □Yes □No □Suspected | | **Mets diagnosed by：**  □Pathology □imaging  □PET-CT □Physical examination | | | | | | **Date of mets (m/d/y):** | | **Location of mets:**  □ Liver □ Lung □ Bone  □ Retroperitoneal lymph □ Peritoneum □ Brain □ Ovary □Others | | |
| **Resectability**:  □Resectable □convertible □Unresectable | | | | | | **Reoperation for mets**:  □ Yes□ No | | | | **Date of surgery for mets**  **(m/d/y):** | | |
| **Name of Surgery:** | | | | | | **Grading of resection**：  □R0 □R1 □R2 | | | | **Other therapies：**  □None □Chemotherapy  □Radiotherapy □Intervention  □ Radiofrequency □Others | | |
| **Postoperative pathology:** | | | | | | | | | | | | |
| **Local recurrence:**  □Yes □No □Suspected | | **LR diagnosed by：**  □Pathology □imaging  □PET-CT □Physical examination | | | | | | **Date of LR (m/d/y):** | | | **Location of LR**:  □ Anastomosis □ Next to bowel □ Lateral □ Incision | |
| **Rectal recurrence classification:**  □Center type □Forward type  □Side type □Backward type | | | **Recurrence subsides in the lateral region:**  □ Left common iliac □ Left external iliac □ Left internal iliac □ Left obturator  □ Right common iliac □ Right external iliac □ Right internal iliac □ Right obturator | | | | | | | | | |
| **Reoperation for LR:**□ Yes□ No | | | | **Date of surgery for LR**  **(m/d/y):** | | | | | **Name of Surgery:** | | | |
| **Grading of resection**：□R0 □R1 □R2 | | | | **Other therapies：**□None □Chemotherapy □Radiotherapy □Intervention  □ Radiofrequency □Others | | | | | | | | |
| **Other information concerning follow-up：** | | | | | | | | | | | | |

| **Follow-up chart 4** | **Follow-up date (m/d/y)** | | | | | | **Follow-up methods:**  □Out-patient clinic □Telephone  □Others_____ | | | | | **After operation:**  ____________ months |
| --- | --- | --- | --- | --- | --- | --- | --- | --- | --- | --- | --- | --- |
| **Post-discharge complications**：  □No □Yes（Description and treatments）： | | | | | **Reoperation:** □Yes □No | | | | | **Reason for reoperation**： | | |
| **Outcomes:**  □Death □Alive with tumor □Alive without tumor | | | | | **Death Causes:**  □Cancer specific  □other causes | | | | | **Death Date(m/d/y):** | | |
| **DFS：** | | | | | **OS：** | | | | | **LRFS：** | | |
| **Physical examination:** □Normal □Abnormal  Description of physical examine: | | | | | | | | **Colonoscopy:** □Normal □Abnormal  Report of coloscopy： | | | | |
| **Blood test**：CEA： ng/mL; CA19-9： ng/mL ; WBC： *10^9^/L; Hb： g/L; PLT： *10^9^/L; | | | | | | | | | | | | |
| **Abdominal imaging:**  □ Normal □ Abnormal (please describe): | | | | | | | | **Chest imaging**：  □ Normal □ Abnormal (please describe): | | | | |
| **Abdominal and pelvic ultrasonography：**  □ Normal □ Abnormal (please describe): | | | | | | | | **Ower extremity venous ultrasonography:**  □ Normal □ Abnormal (please describe): | | | | |
| **Metastasis：**  □Yes □No □Suspected | | **Mets diagnosed by：**  □Pathology □imaging  □PET-CT □Physical examination | | | | | | **Date of mets (m/d/y):** | | **Location of mets:**  □ Liver □ Lung □ Bone  □ Retroperitoneal lymph □ Peritoneum □ Brain □ Ovary □Others | | |
| **Resectability**:  □Resectable □convertible □Unresectable | | | | | | **Reoperation for mets**:  □ Yes□ No | | | | **Date of surgery for mets**  **(m/d/y):** | | |
| **Name of Surgery:** | | | | | | **Grading of resection**：  □R0 □R1 □R2 | | | | **Other therapies：**  □None □Chemotherapy  □Radiotherapy □Intervention  □ Radiofrequency □Others | | |
| **Postoperative pathology:** | | | | | | | | | | | | |
| **Local recurrence:**  □Yes □No □Suspected | | **LR diagnosed by：**  □Pathology □imaging  □PET-CT □Physical examination | | | | | | **Date of LR (m/d/y):** | | | **Location of LR**:  □ Anastomosis □ Next to bowel □ Lateral □ Incision | |
| **Rectal recurrence classification:**  □Center type □Forward type  □Side type □Backward type | | | **Recurrence subsides in the lateral region:**  □ Left common iliac □ Left external iliac □ Left internal iliac □ Left obturator  □ Right common iliac □ Right external iliac □ Right internal iliac □ Right obturator | | | | | | | | | |
| **Reoperation for LR:**□ Yes□ No | | | | **Date of surgery for LR**  **(m/d/y):** | | | | | **Name of Surgery:** | | | |
| **Grading of resection**：□R0 □R1 □R2 | | | | **Other therapies：**□None □Chemotherapy □Radiotherapy □Intervention  □ Radiofrequency □Others | | | | | | | | |
| **Other information concerning follow-up：** | | | | | | | | | | | | |

| **Follow-up chart 5** | **Follow-up date (m/d/y)** | | | | | | **Follow-up methods:**  □Out-patient clinic □Telephone  □Others_____ | | | | | **After operation:**  ____________ months |
| --- | --- | --- | --- | --- | --- | --- | --- | --- | --- | --- | --- | --- |
| **Post-discharge complications**：  □No □Yes（Description and treatments）： | | | | | **Reoperation:** □Yes □No | | | | | **Reason for reoperation**： | | |
| **Outcomes:**  □Death □Alive with tumor □Alive without tumor | | | | | **Death Causes:**  □Cancer specific  □other causes | | | | | **Death Date(m/d/y):** | | |
| **DFS：** | | | | | **OS：** | | | | | **LRFS：** | | |
| **Physical examination:** □Normal □Abnormal  Description of physical examine: | | | | | | | | **Colonoscopy:** □Normal □Abnormal  Report of coloscopy： | | | | |
| **Blood test**：CEA： ng/mL; CA19-9： ng/mL ; WBC： *10^9^/L; Hb： g/L; PLT： *10^9^/L; | | | | | | | | | | | | |
| **Abdominal imaging:**  □ Normal □ Abnormal (please describe): | | | | | | | | **Chest imaging**：  □ Normal □ Abnormal (please describe): | | | | |
| **Abdominal and pelvic ultrasonography：**  □ Normal □ Abnormal (please describe): | | | | | | | | **Ower extremity venous ultrasonography:**  □ Normal □ Abnormal (please describe): | | | | |
| **Metastasis：**  □Yes □No □Suspected | | **Mets diagnosed by：**  □Pathology □imaging  □PET-CT □Physical examination | | | | | | **Date of mets (m/d/y):** | | **Location of mets:**  □ Liver □ Lung □ Bone  □ Retroperitoneal lymph □ Peritoneum □ Brain □ Ovary □Others | | |
| **Resectability**:  □Resectable □convertible □Unresectable | | | | | | **Reoperation for mets**:  □ Yes□ No | | | | **Date of surgery for mets**  **(m/d/y):** | | |
| **Name of Surgery:** | | | | | | **Grading of resection**：  □R0 □R1 □R2 | | | | **Other therapies：**  □None □Chemotherapy  □Radiotherapy □Intervention  □ Radiofrequency □Others | | |
| **Postoperative pathology:** | | | | | | | | | | | | |
| **Local recurrence:**  □Yes □No □Suspected | | **LR diagnosed by：**  □Pathology □imaging  □PET-CT □Physical examination | | | | | | **Date of LR (m/d/y):** | | | **Location of LR**:  □ Anastomosis □ Next to bowel □ Lateral □ Incision | |
| **Rectal recurrence classification:**  □Center type □Forward type  □Side type □Backward type | | | **Recurrence subsides in the lateral region:**  □ Left common iliac □ Left external iliac □ Left internal iliac □ Left obturator  □ Right common iliac □ Right external iliac □ Right internal iliac □ Right obturator | | | | | | | | | |
| **Reoperation for LR:**□ Yes□ No | | | | **Date of surgery for LR**  **(m/d/y):** | | | | | **Name of Surgery:** | | | |
| **Grading of resection**：□R0 □R1 □R2 | | | | **Other therapies：**□None □Chemotherapy □Radiotherapy □Intervention  □ Radiofrequency □Others | | | | | | | | |
| **Other information concerning follow-up：** | | | | | | | | | | | | |

| **Follow-up chart 6** | **Follow-up date (m/d/y)** | | | | | | **Follow-up methods:**  □Out-patient clinic □Telephone  □Others_____ | | | | | **After operation:**  ____________ months |
| --- | --- | --- | --- | --- | --- | --- | --- | --- | --- | --- | --- | --- |
| **Post-discharge complications**：  □No □Yes（Description and treatments）： | | | | | **Reoperation:** □Yes □No | | | | | **Reason for reoperation**： | | |
| **Outcomes:**  □Death □Alive with tumor □Alive without tumor | | | | | **Death Causes:**  □Cancer specific  □other causes | | | | | **Death Date(m/d/y):** | | |
| **DFS：** | | | | | **OS：** | | | | | **LRFS：** | | |
| **Physical examination:** □Normal □Abnormal  Description of physical examine: | | | | | | | | **Colonoscopy:** □Normal □Abnormal  Report of coloscopy： | | | | |
| **Blood test**：CEA： ng/mL; CA19-9： ng/mL ; WBC： *10^9^/L; Hb： g/L; PLT： *10^9^/L; | | | | | | | | | | | | |
| **Abdominal imaging:**  □ Normal □ Abnormal (please describe): | | | | | | | | **Chest imaging**：  □ Normal □ Abnormal (please describe): | | | | |
| **Abdominal and pelvic ultrasonography：**  □ Normal □ Abnormal (please describe): | | | | | | | | **Ower extremity venous ultrasonography:**  □ Normal □ Abnormal (please describe): | | | | |
| **Metastasis：**  □Yes □No □Suspected | | **Mets diagnosed by：**  □Pathology □imaging  □PET-CT □Physical examination | | | | | | **Date of mets (m/d/y):** | | **Location of mets:**  □ Liver □ Lung □ Bone  □ Retroperitoneal lymph □ Peritoneum □ Brain □ Ovary □Others | | |
| **Resectability**:  □Resectable □convertible □Unresectable | | | | | | **Reoperation for mets**:  □ Yes□ No | | | | **Date of surgery for mets**  **(m/d/y):** | | |
| **Name of Surgery:** | | | | | | **Grading of resection**：  □R0 □R1 □R2 | | | | **Other therapies：**  □None □Chemotherapy  □Radiotherapy □Intervention  □ Radiofrequency □Others | | |
| **Postoperative pathology:** | | | | | | | | | | | | |
| **Local recurrence:**  □Yes □No □Suspected | | **LR diagnosed by：**  □Pathology □imaging  □PET-CT □Physical examination | | | | | | **Date of LR (m/d/y):** | | | **Location of LR**:  □ Anastomosis □ Next to bowel □ Lateral □ Incision | |
| **Rectal recurrence classification:**  □Center type □Forward type  □Side type □Backward type | | | **Recurrence subsides in the lateral region:**  □ Left common iliac □ Left external iliac □ Left internal iliac □ Left obturator  □ Right common iliac □ Right external iliac □ Right internal iliac □ Right obturator | | | | | | | | | |
| **Reoperation for LR:**□ Yes□ No | | | | **Date of surgery for LR**  **(m/d/y):** | | | | | **Name of Surgery:** | | | |
| **Grading of resection**：□R0 □R1 □R2 | | | | **Other therapies：**□None □Chemotherapy □Radiotherapy □Intervention  □ Radiofrequency □Others | | | | | | | | |
| **Other information concerning follow-up：** | | | | | | | | | | | | |

| **Follow-up chart 7** | **Follow-up date (m/d/y)** | | | | | | **Follow-up methods:**  □Out-patient clinic □Telephone  □Others_____ | | | | | **After operation:**  ____________ months |
| --- | --- | --- | --- | --- | --- | --- | --- | --- | --- | --- | --- | --- |
| **Post-discharge complications**：  □No □Yes（Description and treatments）： | | | | | **Reoperation:** □Yes □No | | | | | **Reason for reoperation**： | | |
| **Outcomes:**  □Death □Alive with tumor □Alive without tumor | | | | | **Death Causes:**  □Cancer specific  □other causes | | | | | **Death Date(m/d/y):** | | |
| **DFS：** | | | | | **OS：** | | | | | **LRFS：** | | |
| **Physical examination:** □Normal □Abnormal  Description of physical examine: | | | | | | | | **Colonoscopy:** □Normal □Abnormal  Report of coloscopy： | | | | |
| **Blood test**：CEA： ng/mL; CA19-9： ng/mL ; WBC： *10^9^/L; Hb： g/L; PLT： *10^9^/L; | | | | | | | | | | | | |
| **Abdominal imaging:**  □ Normal □ Abnormal (please describe): | | | | | | | | **Chest imaging**：  □ Normal □ Abnormal (please describe): | | | | |
| **Abdominal and pelvic ultrasonography：**  □ Normal □ Abnormal (please describe): | | | | | | | | **Ower extremity venous ultrasonography:**  □ Normal □ Abnormal (please describe): | | | | |
| **Metastasis：**  □Yes □No □Suspected | | **Mets diagnosed by：**  □Pathology □imaging  □PET-CT □Physical examination | | | | | | **Date of mets (m/d/y):** | | **Location of mets:**  □ Liver □ Lung □ Bone  □ Retroperitoneal lymph □ Peritoneum □ Brain □ Ovary □Others | | |
| **Resectability**:  □Resectable □convertible □Unresectable | | | | | | **Reoperation for mets**:  □ Yes□ No | | | | **Date of surgery for mets**  **(m/d/y):** | | |
| **Name of Surgery:** | | | | | | **Grading of resection**：  □R0 □R1 □R2 | | | | **Other therapies：**  □None □Chemotherapy  □Radiotherapy □Intervention  □ Radiofrequency □Others | | |
| **Postoperative pathology:** | | | | | | | | | | | | |
| **Local recurrence:**  □Yes □No □Suspected | | **LR diagnosed by：**  □Pathology □imaging  □PET-CT □Physical examination | | | | | | **Date of LR (m/d/y):** | | | **Location of LR**:  □ Anastomosis □ Next to bowel □ Lateral □ Incision | |
| **Rectal recurrence classification:**  □Center type □Forward type  □Side type □Backward type | | | **Recurrence subsides in the lateral region:**  □ Left common iliac □ Left external iliac □ Left internal iliac □ Left obturator  □ Right common iliac □ Right external iliac □ Right internal iliac □ Right obturator | | | | | | | | | |
| **Reoperation for LR:**□ Yes□ No | | | | **Date of surgery for LR**  **(m/d/y):** | | | | | **Name of Surgery:** | | | |
| **Grading of resection**：□R0 □R1 □R2 | | | | **Other therapies：**□None □Chemotherapy □Radiotherapy □Intervention  □ Radiofrequency □Others | | | | | | | | |
| **Other information concerning follow-up：** | | | | | | | | | | | | |

| **Follow-up chart 8** | **Follow-up date (m/d/y)** | | | | | | **Follow-up methods:**  □Out-patient clinic □Telephone  □Others_____ | | | | | **After operation:**  ____________ months |
| --- | --- | --- | --- | --- | --- | --- | --- | --- | --- | --- | --- | --- |
| **Post-discharge complications**：  □No □Yes（Description and treatments）： | | | | | **Reoperation:** □Yes □No | | | | | **Reason for reoperation**： | | |
| **Outcomes:**  □Death □Alive with tumor □Alive without tumor | | | | | **Death Causes:**  □Cancer specific  □other causes | | | | | **Death Date(m/d/y):** | | |
| **DFS：** | | | | | **OS：** | | | | | **LRFS：** | | |
| **Physical examination:** □Normal □Abnormal  Description of physical examine: | | | | | | | | **Colonoscopy:** □Normal □Abnormal  Report of coloscopy： | | | | |
| **Blood test**：CEA： ng/mL; CA19-9： ng/mL ; WBC： *10^9^/L; Hb： g/L; PLT： *10^9^/L; | | | | | | | | | | | | |
| **Abdominal imaging:**  □ Normal □ Abnormal (please describe): | | | | | | | | **Chest imaging**：  □ Normal □ Abnormal (please describe): | | | | |
| **Abdominal and pelvic ultrasonography：**  □ Normal □ Abnormal (please describe): | | | | | | | | **Ower extremity venous ultrasonography:**  □ Normal □ Abnormal (please describe): | | | | |
| **Metastasis：**  □Yes □No □Suspected | | **Mets diagnosed by：**  □Pathology □imaging  □PET-CT □Physical examination | | | | | | **Date of mets (m/d/y):** | | **Location of mets:**  □ Liver □ Lung □ Bone  □ Retroperitoneal lymph □ Peritoneum □ Brain □ Ovary □Others | | |
| **Resectability**:  □Resectable □convertible □Unresectable | | | | | | **Reoperation for mets**:  □ Yes□ No | | | | **Date of surgery for mets**  **(m/d/y):** | | |
| **Name of Surgery:** | | | | | | **Grading of resection**：  □R0 □R1 □R2 | | | | **Other therapies：**  □None □Chemotherapy  □Radiotherapy □Intervention  □ Radiofrequency □Others | | |
| **Postoperative pathology:** | | | | | | | | | | | | |
| **Local recurrence:**  □Yes □No □Suspected | | **LR diagnosed by：**  □Pathology □imaging  □PET-CT □Physical examination | | | | | | **Date of LR (m/d/y):** | | | **Location of LR**:  □ Anastomosis □ Next to bowel □ Lateral □ Incision | |
| **Rectal recurrence classification:**  □Center type □Forward type  □Side type □Backward type | | | **Recurrence subsides in the lateral region:**  □ Left common iliac □ Left external iliac □ Left internal iliac □ Left obturator  □ Right common iliac □ Right external iliac □ Right internal iliac □ Right obturator | | | | | | | | | |
| **Reoperation for LR:**□ Yes□ No | | | | **Date of surgery for LR**  **(m/d/y):** | | | | | **Name of Surgery:** | | | |
| **Grading of resection**：□R0 □R1 □R2 | | | | **Other therapies：**□None □Chemotherapy □Radiotherapy □Intervention  □ Radiofrequency □Others | | | | | | | | |
| **Other information concerning follow-up：** | | | | | | | | | | | | |

**Long-term endpoints (Completed at the end of the study)**

**Deadline for follow-up(month/day/year)**:

Patient’s status:

□Tumor-free survival □Survival with tumor □Survival with a second primary tumor

□Death (□Died of rectal cancer □Died of complications □Died of second primary cancer □Died of other causes________)

□Lost to follow-up

End point event (recurrence) time (m/d/y): __________________

Local-regional recurrence: □No □Yes (site): __________________

Metastases: □No □Yes (site): __________________

Simultaneous recurrence: ____________+____________+____________

**End point event judgment basis:**

End event type:

□ Local recurrence after program completion

□ Metastasis after program completion

□ Metastasis during radiotherapy and chemotherapy

□ Local progression of the tumor reached the unresectable standard before completion of the program

□ Local recurrence or metastasis occurred in cCR patients who chose Watch & Wait

Pathology confirmed: □Yes □No Report: ________ Confirmed date (m/d/y): __________________

Clinical diagnosis (Diagnostic method: ________ ) Confirmed date (m/d/y): __________________

Second primary tumor status:

Site: ________ Time:________ Treatment:________ Efficacy:________

Retreatment after failure:（Time / Retreatment site / Treatment / Efficacy）

Recurrent chemotherapy

Recurrent surgery

Recurrent Radiotherapy

OS(month) CSS(month)

DFS(month) PFS(month)

LC(month) LRC(month)
