# Supplementary material for: Comparison of the pathological response to 2 or 4 cycles of neoadjuvant CAPOX in II/III rectal cancer patients with low/intermediate risks: study protocol for a prospective, non-inferior, randomized control trial (COPEC trial)
Source: Trials. 2023 Jun 13;24:397. doi: 10.1186/s13063-023-07405-x (PMC10262432; doi:10.1186/s13063-023-07405-x)
Supplement: Supplementary file 5 — Additional file 5. Schedule Of Enrolment, Interventions, And Assessments. [file 13063_2023_7405_MOESM5_ESM.docx]

Table. Schedule Of Enrolment, Interventions, And Assessments

|  | | | STUDY PERIOD | | |
| --- | --- | --- | --- | --- | --- |
| TIMEPOINT | Enrolment | Allocation | | Operation | Follow-up |
| ENROLMENT |  |  | |  |  |
| Eligibility screen | 2021.8-2024.12 |  | |  |  |
| Informed consent | At enrolment |  | |  |  |
| Randomization | At enrolment |  | |  |  |
| INTERVENTIONS |  |  | |  |  |
| 2 cycles group |  | 1.5 months after enrolment | |  |  |
| 4 cycles group |  | 3 months after enrolment | |  |  |
| ASSESSMENTS |  |  | |  |  |
| MRI\CT\CEA\ Symptoms | Within 1 months before enrolment |  | |  |  |
| mrTRG |  | 2 cycles group: before the operation  4cycles group: after 2 cycles and before the operation | |  |  |
| pTRG |  |  | | 3 weeks after operation |  |
| Quality of life/CT/CEA |  |  | |  | Before surgery: each cycle of neoadjuvant therapy (QOL and CEA); after 2 or 4 cycles of neoadjuvant therapy (CT)  After surgery: 3 months, every 6 months in the first 5 years and every year in subsequent years |

MRI magnetic resonance imaging; CT computed tomography; CEA carcino embryonic antigen; mrTRG magnetic resonance tumor regression grade; pTRG, pathological tumour regression grade; QOL quality of life
