# Supplementary material for: Comparison of the pathological response to 2 or 4 cycles of neoadjuvant CAPOX in II/III rectal cancer patients with low/intermediate risks: study protocol for a prospective, non-inferior, randomized control trial (COPEC trial)
Source: Trials. 2023 Jun 13;24:397. doi: 10.1186/s13063-023-07405-x (PMC10262432; doi:10.1186/s13063-023-07405-x)
Supplement: Supplementary file 6 — Additional file 6. Informed Consent. [file 13063_2023_7405_MOESM6_ESM.pdf]

Comparison of the pathological effect for low/intermediate-risk II/III rectal cancer a prospective non-inferior randomized control trial (COPEC trial)

Informed Consent

We invite you to participate in the " Comparison of the pathological effect for low/intermediate-risk II/III rectal cancer a prospective non-inferior randomized control trial (COPEC trial)". This study will be conducted in more than ten large grade A general/specialized hospitals nationwide, and it is estimated that 554 subjects will voluntarily participate in the study.

1. Why is this study conducted?

Neoadjuvant chemoradiotherapy (NCRT) has progressively been accepted as an additional treatment for rectal cancer besides surgical resection. Many previous large randomized controlled trials (RCTs) have shown that NCRT can effectively reduce the local recurrence rate after surgery and is recommended as a priority standard treatment for stage II/III rectal cancer by National Comprehensive Cancer Network (NCCN) guidelines, However, despite reducing the local recurrence rate, NCRT has not been proved with improved overall survival and may also bring radio-related adverse effects. Along with the rapid development of NCRT, the popularization of the concept of TME has further reduced local recurrence, with reported local recurrence rates as low as 5-15%. Local recurrence is no longer the main factor leading to tumor recurrence and death after radical resection.

With the control of local recurrence, distant metastasis, mainly liver and lung metastasis, has gradually become the main recurrence mode affecting the prognosis of rectal cancer.

Our previous prospective stratified randomized controlled study divided stage II/III rectal cancer into high-risk and intermediate-low risk groups according to 5 dimensions (external invasion >5mm, lymph node >8mm, mesorectal fascia (MRF) (+), low anterior wall T3, peripheral growth). The study showed that the local recurrence rate of rectal cancer in the intermediate-low risk group was extremely low, with a 3-year cumulative local recurrence rate of 3%, indicating that at least part of patients with intermediate-low risk were less likely to benefit from radiotherapy, which was also similar to other studies. At the same time, radiotherapy did not bring a significant 5-year survival benefit in either group. Therefore, a consensus has been reached that patients with low- and intermediate-risk stage II/III rectal cancer could consider dispensing with preoperative radiotherapy, and NCT alone might achieve an accepted local control.

Although current studies suggest that NCT alone may benefit patients with low- and intermediate-risk stage II/III rectal cancer, there is still a lack of more detailed stratification and evidence for pathological criteria. For this reason, we have conducted a phase II study that included low- and intermediate-risk stage II/III rectal cancer for NCT alone and found that the proportion of patients with tumor shrinkage and downgrade after only 4 cycles of chemotherapy for low- and intermediate-risk stage II/III rectal cancer was 78.7%, with the pCR rate of 21.3%. In our phase II clinical study,

we also found that the 4 cycles of CAPOX was effective (tumor reduction in long diameter for more than 30% or pTRG grade 0-2), and obvious tumor morphological changes were observed after 2 cycles (duration 6 weeks), with predicted AUC value of 0.862 (0.751, 0.973). And the optimal predictive value reduction of tumor longitudinal diameter was 25.6% with 76.9% sensitivity and 81.3% specificity. These findings suggested that the morphological response of NCT could be better judged at a relatively early stage. Therefore, we tended to hold the view that considerable patients would be able to achieve tumor regression, and the pathological effect would reach Tumor Regression Grade (TRG) 2 or better results after two cycles of NCT.

Based on our previous phase II study, in order to verify that the CAPOX chemotherapy for low- and intermediate-risk stage II/III rectal cancer could achieve a good response judgment after 2 cycles, and obtain the tumor pathological response rate in the early 2 cycles, we intend to conduct a prospective, non-inferior, randomized, controlled study (COPEC trial) to determine the pathological tumor regression grade (pTRG) rate of 2 or 4 cycles of NCT in low- and intermediate-risk stage II/III rectal cancer, and explicit the feasibility of early identification of chemotherapy-insensitive population.

2. What do you need to do to participate in the study?

Participants will be divided into 4 cycles of chemotherapy group and 2 cycles of chemotherapy group. According to the grouping conditions, 2 or 4 cycles of preoperative neoadjuvant chemotherapy will be planned respectively. The existing standard regimen is oxaliplatin 130mg/m2, D1, capecitabine 1000mg/m2, D1-14, Q3W. The 2-cycle group received 2 cycles of chemotherapy, which lasted for about 6 weeks, and completed MRI, CT and ultrasound evaluation, and received TME surgery. The 4-cycle group received 2 cycles of chemotherapy, followed by 2 cycles of chemotherapy after completing the mid-term assessment (MRI, CT, sigmoidoscopy), and underwent surgical treatment after completing the preoperative examination. Postoperative adjuvant therapy was received according to pathological and preoperative clinical stages, and routine review was recommended according to existing guidelines.

3. What are the treatment options available?

The current standard treatment for stage II/III rectal cancer is: neoadjuvant chemoradiotherapy + surgery + adjuvant chemotherapy; Direct surgery can be considered for some low-risk patients.

4. Who should not be included in the study?

1) Patients considering lynch syndrome.2) Patients who do not consider metastasis in the initial diagnosis but proved to be distant metastases during the treatment.3) Previously or concurrently suffering from other malignant tumors (including concurrent colon cancer), except for cured skin basal cell carcinoma and cervical carcinoma in situ.4) Pregnant or nursing women.5) Lateral lymph nodes ≥7mm.6) Patients with severe cardiovascular disease and diabetes difficult to control.7) Patients with mental disorders.8)Patients with severe infection.9) Patients undergoing thrombolysis/anticoagulation therapy with bleeding diathesis or coagulation dysfunction, or suffering aneurysm, stroke, transient ischemic attack in the past year.10) Patients with a history of kidney disease, urinary protein, or clinically abnormal renal

function.11) Patients with a history of gastrointestinal fistula, perforation, bleeding, or severe ulcer.12) Patients with severe gastrointestinal diseases that affect the absorption of oral chemotherapy drugs.13) Patients participating in another clinical trial within 4 weeks before treatment.14) Patients pathologically diagnosed with mucinous component or the signet-ring cell carcinoma.

5. What are the risks of participating in the study?

There are risks and discomfort associated with any study. Capecitabine and oxaliplatin can cause some side effects. It may be that some of the risks of using these drugs have not been identified before. Because these chemotherapy drugs can affect your blood cells, your doctor will order a routine blood test. A low blood count can make you vulnerable to infection or bleeding, which can lead to symptoms such as fever, sore throat, nosebleed, bruising, and blood in your urine or stool. Fever is often the first and most common sign of infection. Your doctor may recommend that you take your temperature frequently, especially during the first week after chemotherapy. If you develop a fever or bleeding/bruising, you should tell your doctor immediately. It is not clear whether chemotherapy drugs affect your fertility, so please tell your doctor about your fertility before starting treatment. It is possible for a woman to stop her menstrual cycle, which may be temporary. Common adverse reactions of capecitabine and oxaliplatin include nausea, vomiting, fatigue, poor appetite, diarrhea, abdominal pain, leukopenia/thrombocytopenia, abnormal liver function, skin reactions, and neurotoxicity. Some drugs can be dangerous to take when you are undergoing chemotherapy, or can increase the side effects of chemotherapy. You should tell your research doctor about any medications you take. Your doctor will monitor for these side effects with blood tests and physical examinations, and prescribe antiemetic and other medications as needed. Your study physician will fully explain all risks associated with treatment. If a toxic reaction is believed to be due to the drug or radiation in the study regimen, it may be possible to adjust your chemotherapy and/or change your use of the study drug by temporarily discontinuing, permanently discontinuing, or reducing the dose. If you experience any discomfort, new changes in your condition, or any unexpected conditions during the study period, whether or not related to the study, you should inform your doctor in a timely manner, and he/she will judge and administer appropriate medical treatment. During the study period, you need to visit the hospital on time and do some examinations, which will take up some of your time and may cause trouble or inconvenience to you. In case of any damage caused by participating in this study, the research team will make full efforts in diagnosis and treatment, integrate multiple scientific resources, and provide free online and offline outpatient consultation compensation.

6. What are the possible benefits of participating in the study?

Currently, oxaliplatin and capecitabine, the chemotherapy drugs involved in this study, are reimbursed as class B drugs in the national medical insurance catalog, which will not increase your economic burden. Although a large amount of evidence has shown that neoadjuvant chemoradiotherapy has satisfactory efficacy for rectal cancer, the side effects caused by radiotherapy can be avoided by neoadjuvant chemotherapy adopted in this study, but there are also chemotherapy-related adverse reactions. If your

condition does not respond or side effects occur, you may ask your doctor about possible alternative treatments, or reduction of dosage or withdrawal of medication. If you participate in this study, your benefit may or may not be better than the current standard treatment plan. Even if you participate in this study, the tumor itself may have a risk of relapse, metastasis and even death in the short term.

7. Do I need to pay any fees to participate in the study?

There is no charge for participating in this study, and you are covered by medical insurance. If serious drug-related adverse reactions occur during treatment and are identified by the research unit as being related to this clinical plan and procedure, compensation will be made within the insurance limit.

8. Is personal information confidential?

Your study data will be kept in the hospitals participating in the study, and your medical records will be accessible to researchers, study authorities and ethics review committees. Your personal identity will not be disclosed in any public report on the results of this study. We will make every effort to protect the privacy and personal information of your personal medical data within the law.

9. Do I have to participate in the study?

Participation in this study is completely voluntary. You may refuse to participate in the study or withdraw from the study at any stage of the study without being subjected to discrimination or retaliation, and your medical treatment and rights and interests will not be affected. If you decide to withdraw from the study, please contact your doctor for proper treatment.

Subject declaration: I have read the above introduction to this study, and my researcher has fully explained and explained to me the purpose of this study, the procedures, the possible risks and potential benefits of participating in this study, and answered all my relevant questions. Volunteer to participate in this study.

Subject signature：____________________________ Date：__ __ __ __
